# Antimonate sequestration from aqueous solution using zirconium, iron and zirconium-iron modified biochars

**DOI:** 10.1038/s41598-021-86978-6

**Published:** 2021-04-14

**Authors:** Md. Aminur Rahman, Mohammad Mahmudur Rahman, Md. Mezbaul Bahar, Peter Sanderson, Dane Lamb

**Affiliations:** 1 Global Centre for Environmental Remediation (GCER), The University of Newcastle, Callaghan, Australia; 2 Global Innovation Centre for Advanced Nanomaterials, The University of Newcastle, Advanced Technology Centre - Room 181, University Drive, Callaghan, NSW 2308 Australia; 3 Department of Public Health Engineering (DPHE), Zonal Laboratory, Khulna, 9100 Bangladesh

**Keywords:** Pollution remediation, Environmental chemistry

## Abstract

Antimony (Sb) is increasingly being recognized as an important contaminant due to its various industrial applications and mining operations. Environmental remediation approaches for Sb are still lacking, as is the understanding of Sb environmental chemistry. In this study, biosolid biochar (BSBC) was produced and utilized to remove antimonate (Sb(V)) from aqueous solution. Zirconium (Zr), Zirconium-iron (Zr–Fe) and Fe–O coated BSBC were synthesized for enhancing Sb(V) sorption capacities of BSBC. The combined results of specific surface area, FTIR, SEM–EDS, TEM–EDS, and XPS confirmed that Zr and/or Zr–Fe were successfully coated onto BSBC. The effects of reaction time, pH, initial Sb(V) concentration, adsorbate doses, ionic strength, temperature, and the influence of major competitive co-existing anions and cations on the adsorption of Sb(V) were investigated. The maximum sorption capacity of Zr–O, Zr–Fe, Zr–FeCl_3_, Fe–O, and FeCl_3_ coated BSBC were 66.67, 98.04, 85.47, 39.68, and 31.54 mg/g respectively under acidic conditions. The XPS results revealed redox transformation of Sb(V) species to Sb(III) occurred under oxic conditions, demonstrating the biochar’s ability to behave as an electron shuttle during sorption. The sorption study suggests that Zr–O and Zr–O–Fe coated BSBC could perform as favourable adsorbents for mitigating Sb(V) contaminated waters.

## Introduction

Anthropogenic activities such as mining, smelting and metallurgy, combustion of fossil fuels, production of flame-retardants, catalysis of plastic production, semiconductors, amongst others, are increasingly important sources of antimony (Sb) in the environment^1,2^. Antimony contamination has received growing attention primarily due to its increasing industrial applications and the relatively limited knowledge on environmental toxicity, transformations and fate in the environment^3,4^. In natural waters, Sb exists in both trivalent (Sb(III) and pentavalent (Sb(V) oxidation states. Trivalent Sb mainly exists as Sb(OH)_3_^0^ and SbO(OH)^0^ at pH 2–10, in the form of SbO^+^ and Sb(OH)_2_^+^ at pH < 2, but can also can be present as SbO_2_^−^ and Sb(OH)_4_^+^ at pH > 10.4. Pentavalent Sb exists mainly as Sb(OH)_6_^−^ at pH > 2.7^5,6^. The toxicity and mobility depends on the Sb oxidation state, with Sb(III) exhibiting tenfold greater toxicity than Sb(V)^7–9^. In addition, antimony trioxide (Sb_2_O_3_) has been identified as a carcinogenic to humans (Group 2B) by IARC^10^.

Considering the increasing threat posed by Sb in the environment, several technologies have been trialled to remove excess Sb from natural waters. Remediation technologies to date rely on (ad)sorption, coagulation, electrocoagulation, co-precipitation, electrodeposition and membrane techniques^11^. Due to the expensive operational and maintenance costs involved in such processes, most of these techniques have limitations in removing Sb. The adsorption process has emerged as a promising and viable approach due to its economical nature, high efficiency, simplicity, technical flexibility, and social acceptability^12,13^.

Several adsorbents have been investigated for Sb removal from natural and industrial waters, including multiwalled carbon nanotubes^14^, hydrated ferric oxide supported by calcite sand^15^, graphene^16^, Fe-modified aerobic granules^17^, and Fe–Mn binary oxide^18^. The adsorption capacity of these materials tends to be poor. Only a few sorbents such as metal-loaded Zr(IV), Fe(III) saponified orange waste^19^, zero-valent iron nanoparticles coatings on aluminum and silicon minerals^20,21^, iron oxyhydroxides, zirconium oxide (Zr–O)-carbon nanofibers^13^, reduced graphene oxide/Mn_3_O_4_^22^, MnO_2_ nanofibers^23,24^ , TiO_2_^24^ , and UiO–66NH_2_^25^ have reported promising results for both Sb(III) and Sb(V) adsorption. However, readily available and cost-effective materials are required to remediate Sb from contaminated water.

Recently, biochar has received notable attention as environmentally friendly and effective adsorbents, cost-efficient materials for the remediation treatment of metal(loid)s^26^. Although several studies have documented Sb adsorption to pure minerals^27,28^ and humic substances^29,30^, very few studies of Sb sorption onto biochars have been reported to date^31,32^. Antimonate can strongly bind to Fe(OH)_2_/Fe(OH)_3_, but, the bonding environment is still not clearly resolved^33,34^. Similarly, little is currently known on the Sb sorption by biochars, sorption mechanisms and the possible surface transformations which may occur.

Biochars have been modified to improve the sorption properties for oxyanions such as Sb and As. For instance, one study investigated the elimination of Sb(III) using different metal oxide composites consisting of Fe and Mn^18^. Hydrous Zr–O is known to display ion exchange ability, and specific binding potentiality of Zr(IV) to different oxyanions due to its strong Zr(IV)–O bonds^35^. The excellent performance of Zr-based metal organic frameworks were presented in removing Sb and As from water^25^. Ren et al.^36^ demonstrated the high removal capacity for As using Fe–Zr binary oxide. Being from the same group (group V), As and Sb share some similar properties, but also show contrasting interactions with Fe and organic moeities^30,37^.

In this study, we report the Sb(V) removal with modified and unmodified biochars and potential redox transformations associated with biochar. The objectives of this study were to: (1) synthesise a series of Zr treated biochar, Zr–Fe treated biochar and Fe treated biochar by a co-precipitation method, and (2) evaluate the adsorption performance of Sb(V) in aqueous solution as controlled by solution pH, adsorbate dosage, reaction time, initial concentration, temperature, influence of major coexisting cations and anions, surface charge, and surface area. In addition, we explored the surface transformation of Sb(V) to Sb(III) under oxic conditions with X-ray photoelectron spectroscopy (XPS).

## Results and discussion

### Biochar characterization

The zeta potential of all biochars in the range of pH 2–11 varied between + 25.02 to − 35.54 mV (Table S1 in Supporting Information (SI) section). Increasing pH translated to increasing negative surface charge of pristine biochars. The net surface charge of modified Zr–FeCl_3_BSBC(1:5) and Zr–FeBSBC(1:20) carried positive charge up to pH 5 and 6, whereas Zr–BSBC_6.5,_ Zr–BSBC_12.5_, Fe–BSBC_,_ and FeCl_3_–BSBC carried a positive surface charge at pH < 3. At low pH, the net protonation from the medium was enough to balance the negative charge of all biochars. The iso-electric point was reached at pH 2–6 (Table S1). The physico-chemical characteristics and elemental composition of biochars are provided in Table S2 and Table S3, respectively.

**Figure 1:**
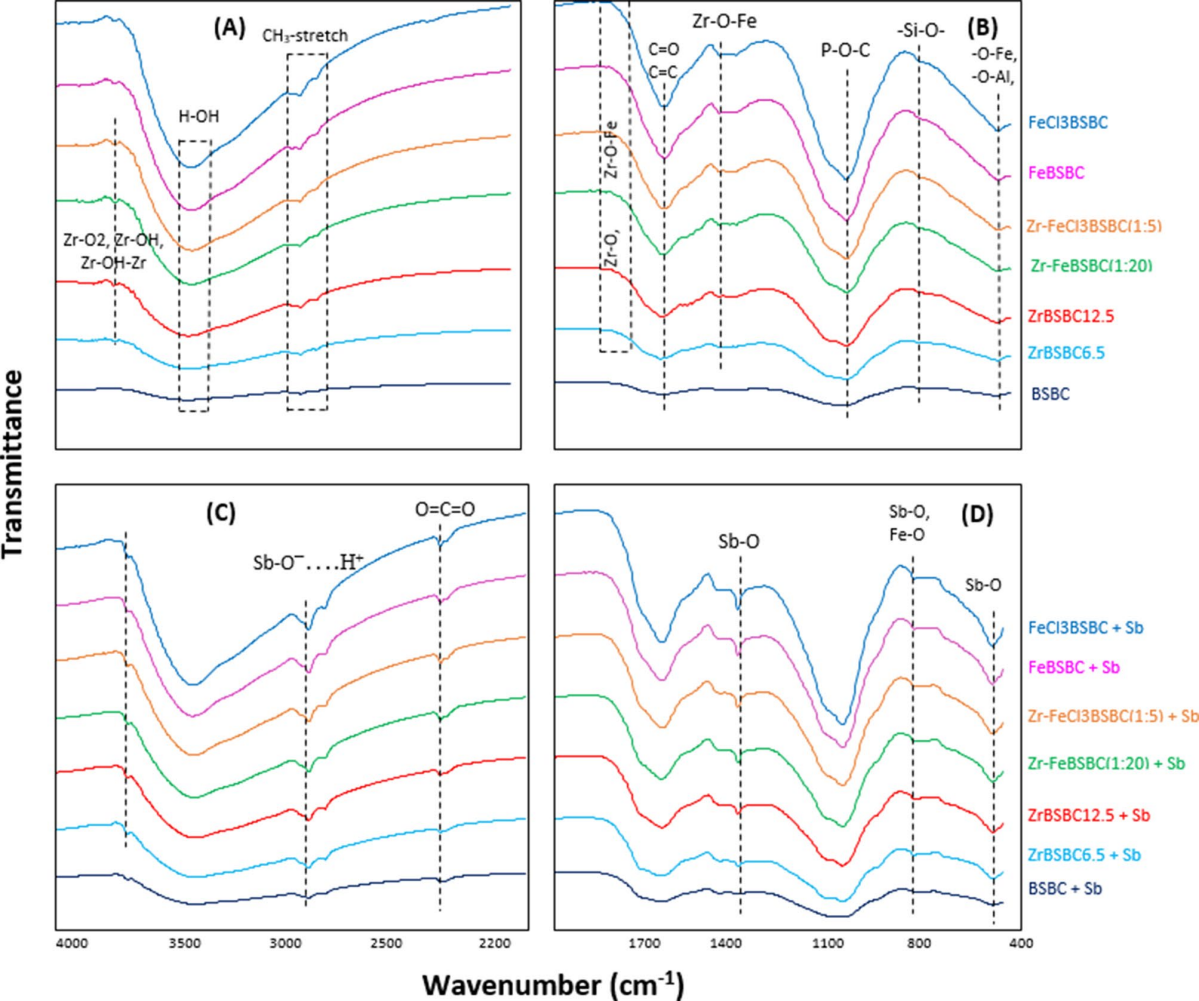
FTIR Spectra of biochars before (**A**, **B**) and after (**C**, **D**) Sb(V)-sorption.

The surface functional groups of modified biochars analyzed by FTIR are presented in Fig. 1. The most prominent, broad and strong bands occurred at around 3400 cm^−1^, corresponding to the stretching and bending vibrations of –OH functional groups of tightly bonded water molecules (Fig. 1A)^31,32,38^. The peak at 1640 cm^−1^ was explained by the deformation of water molecules and indicated physio-sorbed H_2_O on the adsorbent by oxide^32,36^. Previous studies also support this^39,40^. The spectra at around 2900 cm^−1^ could be attributed to CH_3_– stretching which exists in all biochars; similar findings were reported by Vithanage et al.^32^.

A spectral shift of 2915–2960 and 2845–2865 cm^−1^ occurred due to Zr and/or Fe modifications compared to the pristine biochar (Fig. 1A). The two spectrum bands at 3694 and 3791 cm^−1^ (Fig. 1A) ascribed to freely vibrating surface hydroxyl groups can be found in BSBC, FeBSBC and FeCl_3_BSBC. However, after Zr-modification the bands shifted to 3646, 3671, and 3687 cm^−1^, which is responsible for ZrO_2_ monoclinic (Zr–OH) and tetragonal (Zr–OH–Zr) (Fig. 1A) crystalline super structure (tri-bridges OH– groups on Zr^4+^)^41^. Moreover, the band at 3746 cm^−1^ could arise from the SiO_2_ group. The bands 1522, 1541, 1544, 1559, and 1574 cm^−1^ were due to Zr–OH vibrations^42^ found in the Zr and/or Fe-modified BSBC. The Zr and P peaks always overlapped. In addition, peaks at 1315, 1339, 1343, 1397, 1418, and 1420 cm^−1^ (1300–1420 cm^−1^) ascribed to carboxylate groups^36^ were developed in Zr-modified biochars (Fig. 1B). This was due to the deformation vibration of Zr–OH. Samples reacted with Sb(V) demonstrated a new sharp band at 1384 cm^−1^ (Fig. 1C,D) which was not observed in unreacted biochars. The absorption band is attributed to the Sb–O bond. Another distinguishable feature is that the structure of the spectra at 795 cm^−1^ of Sb(V) reacted biochar was sharper than the Sb(V)-unreacted biochar (Fig. 1D).

Figure S1 shows representative SEM images of each of the biochars. Distinct micropores were observable, especially in the unmodified BSBC biochars. Figure S1(i–ii); and B(i–ii) to G(i–ii), represents the morphology and surface characteristics of pristine BSBC and different modified biochars, respectively, before the sorption of Sb(V) at two different magnifications. Similarly, Figure S1(iii), and 2B(iii)–G(iii) describes the morphology and surface texture of Sb-loaded pristine, and Sb-loaded modified biochar-composites, respectively, after sorption of Sb(V) from aqueous solution. The morphology and surface texture of modified biochars contrasted from one another, which were covered by high densities of fine Zr, or Zr–Fe particles. Moreover, some fractions of Zr(IV) were located randomly onto the biochar surface which indicates a heterogeneous coating of these metals occurred during co-precipitation [Figure S1 B(i)–E(i)].

The SEM–EDS spectrum of ZrBSBC_6.5_, ZrBSBC_12.5_, Zr–FeBSBC(1:20) and Zr–FeCl_3_BSBC(1:5) confirmed the presence of Zr in the Zr and/or Zr–Fe-modified biochars at 2.042 keV [Figure S2 B(i)–E(i)]; and Figure S2 A(i), S2 F(i) and S2 G(i) represents the SEM–EDS of pristine BSBC, FeBSBC and FeCl_3_BSBC, respectively. After sorption of Sb(V) onto these adsorbents and the characteristic peaks at 3.604 keV in the EDX spectra [Figure S2 A(ii)–G(ii)], this confirmed the existence of sorbed Sb(V) along with C, O, N, Fe, P, Sb, and Zr. However, SEM–EDS spectra of P (K-line 2.013 keV) and Zr (L-line 2.042 keV) in biochars may not be clearly differentiated due to their very close X-ray energy levels. Thus, overlapping spectra of P and Zr was observed. To overcome this issue, TEM–EDS was performed where the Zr K-line was confirmed at 15.744 keV and could be differentiated from the P K-line. The SEM–EDS analysis also provided evidence that Zr–FeBSBC (1:20) showed substantial wt. (%) distribution of Sb(V) (10.94 wt. %) than other biochars, indicating Zr–FeBSBC (1:20) possessed a high Sb(V) sorption efficiency.

**Figure 2:**
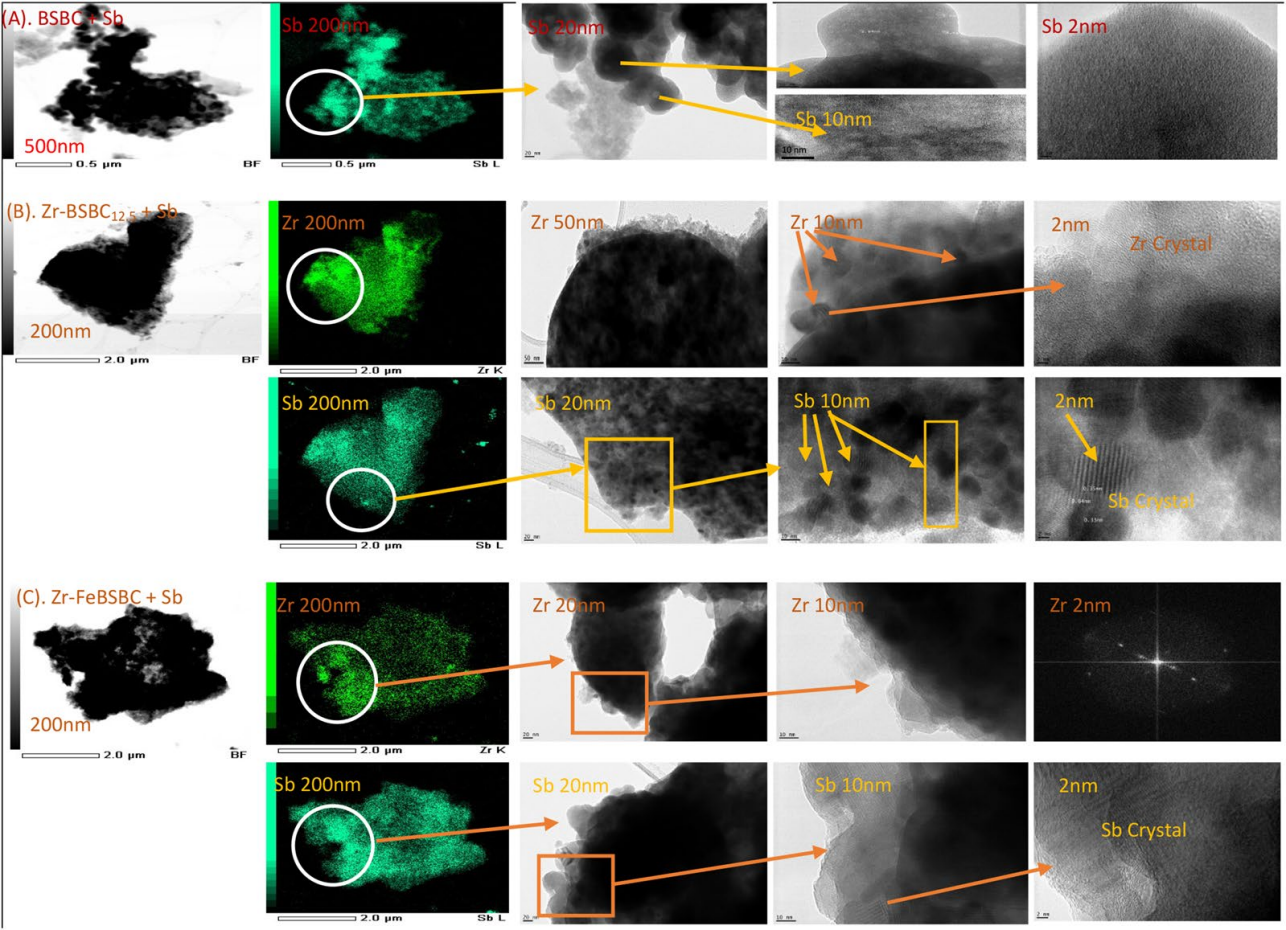
Different magnifications of Sb(V) and Zr onto Sb-loaded (**A**) BSBC, (**B**) ZrBSBC_12.5_, and (**C**) Zr-FeCl_3_BSBC biochars revealed by TEM imaging (Zr and Sb-crystallinity).

The TEM images of pristine BSBC, ZrBSBC_12.5_ and Zr–FeBSBC (1:20) are shown in Figure S3. Results from EDS–TEM elemental mapping indicated that C, O, Ca, P, Si, S, Ca, K, Mg, were the major elements in biochar structure, yet the intensity and brightness of C, O, P and Ca were more noticeable in pristine BSBC (Figure S3A). TEM elemental images also confirmed the presence of Zr and Zr–Fe in Zr–BBSC_12.5_ and Zr–FeBSBC (1:20) (Figure S3C and S3D). The TEM–EDS spectrum of ZrBSBC_12.5_ and Zr–FeBSBC (1:20) confirmed the presence of Zr (K-line) (mass percentage 15.06% and 2.79%, respectively) located heterogeneously on the biochar surfaces, suggesting the procedure successfully coated Zr with biochar at 2.042 keV (L-line) (Figure S4 in SI section). Characteristic Sb spectra were identified characteristics Sb spectra were found after sorption of Sb(V) with BSBC (Sb mass 15.44%), ZrBSBC_12.5_ (Sb mass 3.45%, Zr mass 11.80%) and Zr–FeBSBC(1:20) (Sb mass 5.34%, Zr mass 0.73%) (see Figure S4 in SI Section). However, TEM images demonstrate different forms of non-uniform nanosized Zr and Sb crystals (Fig. 2). The lattice planes could be clearly sighted with a *d*-spacing at ~ 0.35 nm, characteristic of the (111) plane of the Zr-crystalline tetragonal phase^43^(Fig. 2). The formation of Sb crystal features on the biochar surfaces indicates surface modification and transformation of Sb to a possible 3-dimensional feature on the surface.

**Figure 3:**
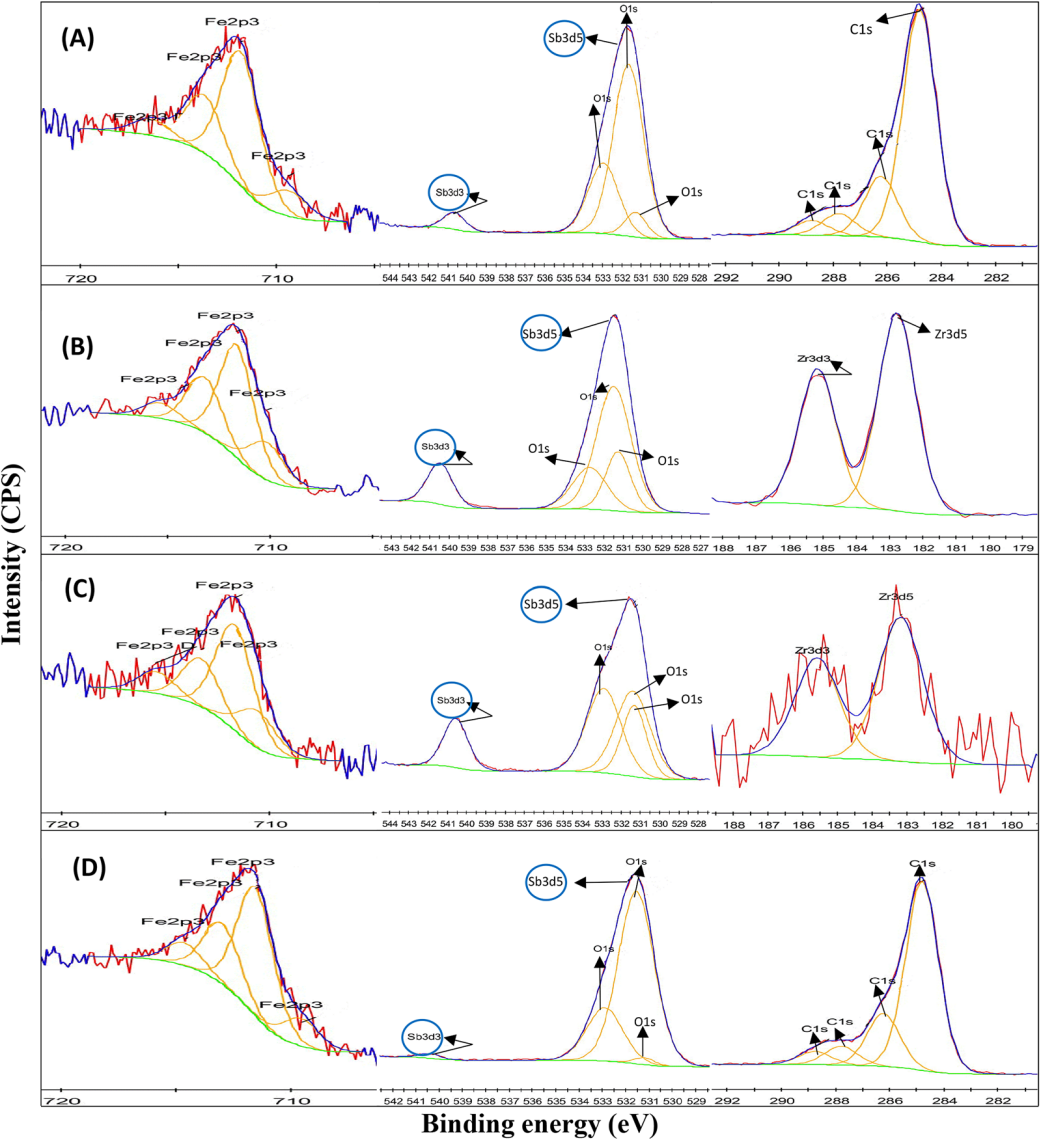
XPS expanded peaks of Sb-loaded BSBC **(A)**, ZrBSBC12.5 **(B)**, Zr-FeBSBC(1:20) **(C)** and FeBSBC **(D)**.

The results from XPS analysis similarly confirmed the transformation of Sb(V) at the surface of all biochars (Fig. 3). The two peaks of Sb 3d3 and Sb 3d5 are located at binding energies of 531.6 and 540.7 eV after sorption of Sb (Fig. 3A) which demonstrates the existence of both Sb(V) and Sb(III) species on the biochar surface. The appearance of two Sb peaks suggests that the reduction of Sb(V) to Sb(III) occurred during sorption by pristine BSBC as well as Zr-modified, Zr–Fe modified and Fe-modified biochars under oxidizing environments. Previous research on As has indicated redox transformations may occur during reaction with pyrolised organic materials, such as the surface of biochars^31^. Kappler et al.^44^ demonstrated that biochar particles under reducing conditions acted as an electron shuttle, resulting in redox transformation of Fe and potentially other redox sensitive elements. The current work indicates, that even under oxidizing conditions, biochar surfaces may promote reductive transformation of Sb during reaction, whether modified with Zr (Fig. 3B) or Fe (Fig. 3D).

The O_ad_/O_latt_ ratio increased from 1.0 to 1.21 and 1.0 to 1.11 from BSBC to ZrBSBC_12.5_ and FeBSC after Sb sorption, respectively. However, this ratio decreased to 0.55 in Zr–FeBSBC(1:20) due to the incorporation of additional Fe association with Zr. In addition, the Fe content decreased from 0.51 to 0.17 at % for ZrBSBC_12.5_ compared to pristine BSBC. Interestingly, the Fe content increased for Zr–Fe (0.51–0.56 at.%) and Fe-modified (0.51–0.66 at.%) biochars compared to pristine biochar. Therefore, the Zr–O, Zr–O–Fe or Fe–O site play significant role for enhanced Sb sorption by Zr–BSBC, Zr–FeBSBC and FeBSBC.

The chemical composition of pristine BSBC, ZrBSBC_12.5_, Zr–FeBSBC (1:20) and FeBSBC after Sb(V) sorption was characterized by XPS. The XPS survey spectrum clearly showed the corresponding peaks to O 1 s (531.65 eV), N 1 s (400.21 eV), C 1 s (284.8 eV), P 2p (133.77 eV), Fe 2p (711.83), and Sb 3d (540.65 eV) (Figure S5). The XPS survey spectra confirmed the successful bonding of Zr onto ZrBSBC_12.5_ (Zr3d5 at 182.76 and Zr 3d3 at 185.45 eV) and Zr–FeBSC (1:20) (Zr 3d5 at 183.17 and Zr 3d3 at 185.36 eV) surface (Fig. 3B,C). The two Zr 3d peaks can be observed at two binding energies and exists in ( +) 4 oxidation state^45^. The peak at approximately 182.76 and 185.36 eV represent to the a Zr–O bonds while the peaks at approximately 182.81 and 185.36 eV correspond to the metallic Zr bonds (Zr–Zr) which was slightly shifted by loading of Sb (Fig. 3B,C)^13^**.**

### Antimonate sorption to biochars

#### Effect of pH

**Figure 4:**
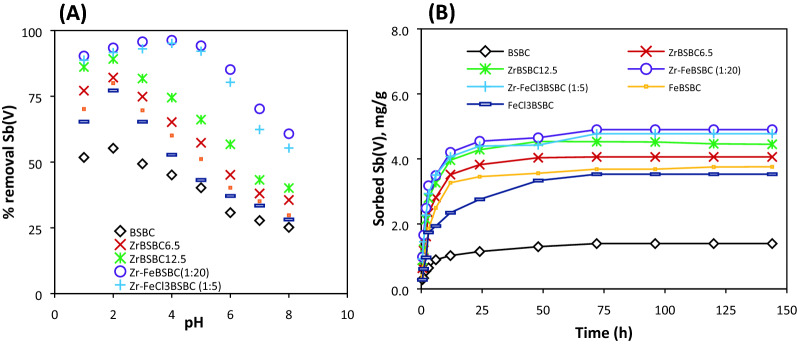
Effect of pH on removal percentage (%) of Sb(V), (**A**) [Initial concentration was 20 mg/L Sb(V), biochar dosage was 4 g/L, temperature was 22 °C] and effect of time on sorption capacity of Sb(V), (**B**) [Initial concentration 5 mg/L for BSBC, and 20 mg/L for ZrBSBC_6.5_, ZrBSBC_12.5_, Zr-FeBSBC(1:20), Zr-FeCl_3_BSBC (1:5), Fe-BSBC and FeCl_3_-BSBC Sb(V), biochar dosage was 4 g/L, temperature was 22 °C].

The highest adsorption (85–96%) appeared at a broader range of pH 2–6 for modified Zr–FeBSBC (1:20) and Zr–FeCl_3_BSBC (1:5), whereas for pristine BSBC the rates were low between pH 3 and 8, which sharply declined at pH 8 (25%) (Fig. 4A).

At pH < 5, the biochar composites behave as weak acids and formed positivity charged surfaces sites. Since most of the metallic oxides/hydroxides demonstrated amphoteric surface characteristics, the Zr/Fe oxides could be protonated at acidic pH by the following reactions:



The Zr/Fe oxides could be deprotonated at basic pH by the following reactions:

Thus the presence of Zr and/or Fe in the Sb(V) solution at different pH shows a buffering effect^46,47^. The adsorption capacity of Sb(V) did not change greatly up to pH 3. This might be due to the presence of undissociated Sb(V) species in the aqueous solution. Moreover, the pHzpc of BSBC, ZrBSBC_6.5_, ZrBSBC_12.5_, Zr–FeBSBC(1:20), Zr–FeCl_3_BSBC(1:5), Fe–BSBC and FeCl_3_–BSBC were 3.6, 3.7, 3.8, 6.2, 5.7, 3.9 and 3.8, (Figure S6), and Sb(V) being in undissociated form at this pH range. This subsequently suggest a likely influence of electrostatic binding. Additionally, zeta potential measurements confirmed net negative biochar surfaces at pH > pH_pzc_ (Figure S6). This explains the decrease in sorption capacity as the pH rises above pH_pzc_.

#### Sorption kinetics

The kinetics data well fitted by the pseudo-second-order kinetics model (Table 1). Both pristine and modified biochars slowly adsorbed Sb(V) from aqueous solutions and reached equilibrium in 72 h (Fig. 4B). Antimonate sorption kinetics to biochars did not fit well with the first-order-kinetics and Elovich models. This is because the calculated *q*_*e*_ and experimentally observed *q*_*e*_ were extremely poor at initial Sb(V) concentration (Table 1 and Supporting Information). However, the kinetic data best fitted with the pseudo-second-order model with *R*^2^ values ≥ 0.99 (Table 1). This indicates the likelihood of chemisorption processes and not purely electrostatic interactions between the adsorbent and adsorbate^48^.

**Table 1:** Kinetic models and fitted parameters for Sb(V) sorption data.

Biochar	q_e-exp_ (mg/g)	Pseudo first-order			Pseudo second-order				Elovich			Intraparticle diffusion			pH
		k_1_ (h^−1^)	q_e-cal_	R^2^	k_2_ (g mg^−1^ h^−1^)	q_e-cal_ (mg/g)	h ( mg/g h)	R^2^	β (mg/g)	α (mg/g.h)	R^2^	k_id_ (g mg^−1^ h^−1/2^)	C (mg/g)	R^2^	
BSBC	1.40	0.042	0.96	0.89	0.19	1.43	0.39	0.99	0.42	0.21	0.97	0.10	0.48	0.82	2
ZrBSBC6.5	4.06	0.065	1.50	0.89	0.10	4.15	1.75	0.99	1.56	0.57	0.90	0.26	1.65	0.70	2
ZrBSBC12.5	4.45	0.031	0.96	0.44	0.12	4.55	2.54	0.99	1.89	0.61	0.88	0.27	1.99	0.67	2
Zr–FeBSBC (1:20)	4.91	0.046	1.84	0.88	0.09	4.98	2.31	0.99	2.03	0.62	0.92	0.27	2.24	0.70	4
Zr–FeCl3BSBC (1:5)	4.78	0.052	1.96	0.88	0.08	4.86	2.03	0.99	2.22	0.61	0.91	0.28	2.10	0.70	4
FeBSBC	3.76	0.046	2.05	0.94	0.07	3.85	1.05	0.99	1.10	0.60	0.90	0.26	1.25	0.71	3
FeCl3BSBC	3.53	0.063	2.27	0.89	0.05	3.69	0.67	0.99	0.78	0.60	0.97	0.27	0.91	0.83	2

In this study, the initial linear part of the curve described the surface diffusion and the curve did not intersect through the origin (C ≠ 0) (Figure S7) suggesting that intra-particle diffusion was not the only rate controlling phase but more than one process controls the sorption. Here, Sb(V) uptake was observed in apparent two phases: a sharper linear component attributed to Sb(V) diffusion of Sb(V) species to ZrBSBC_6.5_, ZrBSBC_12.5_, Zr–FeBSBC (1:20), Zr–FeCl_3_ (1:5), FeBSBC and FeCl_3_BSBC through boundary layer diffusion, subsequently followed by intra-particle diffusion (Figure S7).

#### Sorption isotherms

**Figure 5:**
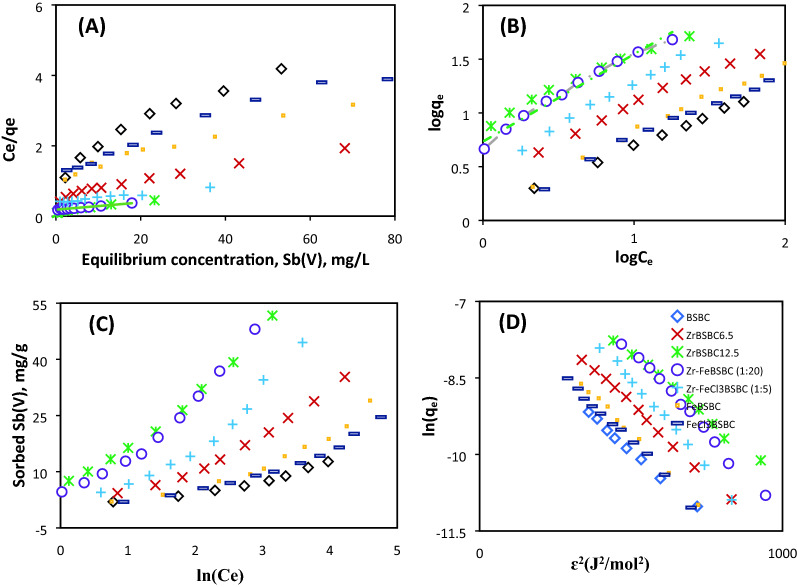
Isotherm model Langmuir (**A**), Freundlich (**B**), Temkin (**C**), and D–R (**D**) (Initial Sb(V) concentration was 5–250 mg/L, biochar density was 4 g/L at 22 °C).

The adsorption of Sb(V) by all modified biochars increased rapidly in the concentration range of 1–10 mg L^−1^ followed by a gradual increase thereafter (Fig. 5). Langmuir, Freundlich, Temkin and Dubinin–Radushkevich models were utilized to fit the experimental data (Table 2 and Fig. 5A–D). Despite the fact that all isotherms fitted well, the Freundlich and Dubinin–Radushkevich models reproduced Sb(V) sorption data overall to highest extent (*R*^2^ ≥ 0.99). This indicates that multilayer sorption was a potential sorption process mechanism for Sb(V) (Table 2 and Fig. 5B,D). Freundlich constant *K*_*F*_ values spanned between 0.49 and 8.04 for all Sb(V) isotherm models with a slightly higher *K*_*F*_ obtained for ZrBSBC_12.5_ than Zr–FeBSBC (1:20), respectively (Table 2). The sorption intensity or heterogeneity of a sorbent surface is indicated by the 1/n that reflects deviance from linearity. The 1/n values were in the order of Zr–FeBSBC (1:20) > Zr–FeCl_3_BSBC (1:5) > Fe–BSBC > Zr–BSBC_6.5_ > FeCl_3_–BSBC > ZrBSBC_12.5_ > BSBC; and spanned 0.583–0.813. This suggested that sorption process was favourable and chemical in nature belongs to the batch experimental conditions (Table 2)^31,49^.

**Table 2:** Sorption isotherm models and best-fit parameters for Sb(V) sorption data.

Biochar	q_exp_ (mg/g)	Langmuir model parameters					Freundlich model parameters				Temkin model parameters			Dubinin–Radushkevich model parameters				pH
		q_cal_ (mg/g)	q_m_ (mg/g)	K_L_ (L mg^−1^)	R_L_	R^2^	q_cal_ (mg/g)	K_F_ (g mg^−1^ h^−1^)	1/n	R^2^	b (J/mol)	A (L/g)	R^2^	q_m_ (mg/g)	E (kJ mol^−1^)	β	R^2^	
BSBC	12.71	12.07	17.54	0.041	0.18–0.69	0.95	12.90	1.27	0.584	0.99	731.6	0.56	0.93	80.23	9.80	5.2 × 10^−3^	0.99	2
ZrBSBC_6.5_	35.31	34.10	46.95	0.039	0.11–0.71	0.98	39.92	2.719	0.636	0.99	261	0.46	0.96	253.26	9.36	5.7 × 10^−3^	0.99	2
ZrBSBC_12.5_	51.68	49.45	66.67	0.123	0.03–0.44	0.97	57.83	8.039	0.627	0.98	177.45	1.23	0.98	508.96	9.90	5.1 × 10^−3^	0.99	2
Zr–FeBSBC (1:20)	47.12	41.60	− 31.8	− 0.033	1.52–0.16	0.54	33.89	0.910	1.271	0.92	153.94	0.39	0.63	4395	6.93	1.0 × 10^−2^	0.90	2
	48.81	47.07	− 60.61	− 0.037	4.0–0.14	0.67	42.58	1.986	1.248	0.97	116.26	0.49	0.81	3202	7.58	8.7 × 10^−3^	0.96	3
	48.04	48.84	98.04	0.055	0.077–0.63	0.96	56.56	5.41	0.8128	0.98	162.64	1.01	0.96	1120	9.77	6.5 × 10^−3^	0.99	4
Zr–FeCl_3_BSBC (1:5)	38.80	36.40	70.42	0.011	0.89–0.29	0.96	31.43	1.633	0.796	0.99	251.55	0.42	0.89	456	8.45	7.0 × 10^−3^	0.99	2
	44.16	37.64	125	0.014	0.87–0.25	0.49	39.20	1.929	0.883	0.99	173.58	0.37	0.86	644	8.45	7.0 × 10^−3^	0.98	3
	44.49	44.47	85.47	0.029	0.13–0.76	0.97	50.08	3.12	0.772	0.99	185.73	0.54	0.93	727	9.57	6.8 × 10^−3^	0.99	4
	47.36	35.44	112.36	0.017	0.85–0.21	0.20	35.81	2.139	0.857	0.94	229.9	0.51	0.82	274	9.62	5.4 × 10^−3^	0.93	5
FeBSBC	39.80	32.91	120.48	0.006	0.93–0.41	0.35	32.16	1.13	0.833	0.99	260.03	0.32	0.76	325	8.45	7.0 × 10^−3^	0.98	2
	28.98	26.51	39.68	0.02	0.18–0.83	0.95	30.29	1.36	0.6739	0.99	365.14	0.35	0.91	181	8.98	6.2 × 10^−3^	0.99	3
	41.26	36.53	49.26	0.071	0.006–0.57	0.91	43.39	4.684	0.603	0.88	238.79	0.86	0.93	444	9.21	5.9 × 10^−3^	0.93	4
FeCl_3_BSBC	41.76	34.67	270.27	0.003	0.96–0.57	0.21	35.16	1.0139	0.9447	0.99	216.26	0.31	0.80	799	7.71	8.4 × 10^−3^	0.99	2
	24.60	22.66	31.54	0.022	0.17–0.81	0.95	26.08	1.313	0.6279	0.98	432.41	0.34	0.92	135	9.20	5.9 × 10^−3^	0.99	3

The maximum Sb(V) sorption capacities (*q*_*m*_) of biochars followed the order Zr–FeBSBC (1:20) > Zr–FeCl_3_BSBC (1:5) > ZrBSBC_12.5_ > ZrBSBC_6.5_ > FeBSBC > FeCl_3_BSBC > BSBC. The maximum sorption of Sb(V) observed for Zr–FeBSBC (1:20), Zr–FeCl_3_BSBC (1:5), ZrBSBC_12.5_, (98.04, 85.47 and 66.67 mg g^−1^) followed by ZrBSBC_6.5_, FeBSBC, FeCl_3_BSBC, BSBC, (46.95, 39.68, 31.54, and 17.54 mg g^1^), respectively (Fig. 5A and Table 2).

The *R*^2^ value for the Temkin model of BSBC was 0.93. For modified biochars *R*^2^ranged from 0.91–0.98 (Table 2 and Fig. 5C). In the Dubinin–Radushkevich (D–R) model, *R*^2^ values were 0.99 (Fig. 5D and Table 2) for pristine and modified biochars, and the higher theoretical sorption of modified biochars were ascribed to its greater micro-porosity and reduced pore diameter. This outcome agreed with the greater SSA of the modified biochars. The bonding energy *E* (kJ mol^−1^) provides indirect data on the sorption mechanism, whether physical or chemical in nature. The calculated values between 8.57–9.20 kJ mol^−1^ indicate that the sorption system takes place chemically (chemisorption); values less than 8 kJ mol^−1^ indicate the system proceeds physically^50^.

#### Effect of major anions, major cations and ionic strength

The widely occurring anions such as SO_4_^2−^, PO_4_^3−^ and CO_3_^2−^ have been revealed to exhibit different effects on the adsorption of Sb^17^. The Cl^−^, NO_3_^−^, and SO_4_^2−^ did not pose any significant effect on Sb(V) sequestration because they have very minor affinities (between 3–6%) (Figure S8A). Carbonate showed little effect on the sorption of Sb(V) even at 1.0 M. The sorption capacity of Sb(V) decreased slightly (up to 12%) from 5.13 to 4.45 mg g^−1^ (96.44–84.07%) due to the presence of CO_3_^2^. It is possibly because the Cl^−^, NO_3_^−^, CO_3_^2−^, SO_4_^2−^ anions could mainly form outer-sphere complexes with biochar and thus affected Sb(V) sorption to minimal extent^51^. Analogously, much more significant retardation of PO_4_^3−^ on Sb(V) sorption was observed, and the sorption capacity decreased (27%) from 4.73 to 3.46 mg g^−1^ (94.86–71.31%) (Figure S8A) even at 0.01 M PO_4_^3−^. The impact of PO_4_^3−^ was particularly evident in the Zr–FeBSBC (1:20), which similarly indicates a specific sorption mechanism in this adsorbent. These results were consistent with previous studies on the Sb(V) adsorption onto Ce(III)-doped Fe_3_O_4_ particles^46^, magnetic sludge particles^52^ and La-doped magnetic biochars^53^. In addition, PO_4_^3−^ may undergo inner-sphere complexation with oxy-hydroxide compounds and compete with the same sorption sites of the biochar composites. The influence of interfering major cations such as Na^+^, K^+^, Mg^2+^, and Ca^2+^ of Sb(V) revealed that no notable changes in Sb(V) sorption (Figure S8B). In this study, a higher ionic strength significantly decreased the sorption capacity by 10.85% (3.37–3.04 mg/g), 19.23% (4.73–3.82 mg/g), 11.88% (5.05–4.45 mg/g), 6.35% (5.35–5.01 mg/g), 11.71% (5.12–4.52 mg/g), 17.18% (4.54–3.76 mg/g) and 14.85% (4.85–4.13 mg/g) for BSBC, ZrBSBC_6.5_, ZrBSBC_12.5_, Zr–FeBSBC(1:20), Zr–FeCl_3_BSBC(1:5), FeBSBC, and FeCl_3_BSBC, respectively, when NaNO_3_ concentrations increased from 0.01–1.0 M (Figure S9A). At high concentrations, the NO_3_^−^ anion may compete with Sb(OH)_6_^−^ for the available sites on the biochar surface, and reduce Sb(V) reaction with the biochar^54,55^. However, as shown below, NO3^−^ did not influence Sb(V) sorption. Secondly, Sb(V) exists mainly as highly polymerised hydroxyl-nitro complexes or colloidal hydrous oxides in the presence of high NO_3_^−^ concentrations^56^. In this research, 0.01 M NaNO_3_^−^ is considered as an ideal background electrolyte.

#### Chemistry of Sb(V) binding mechanisms onto biochars

Antimonate exists in solution predominantly as an anionic species and thus it is expected to bind to biochars largely via ion exchange and ligand exchange mechanisms. Broadly speaking, Sb(V) removal from an aqueous solution via sorption onto Zr–BSBC, Zr–FeBSBC and FeBSBC may be due to one or more factors such as: (1) electrostatic attraction, (2) nodule formation through hydrogen bonding, and (3) surface complexation or ligand exchange. Only at a pH < 2 does Sb(V) form positively charged species to any significant degree. Also, it can be assumed that at pH < 4, the biochar composites should behave as weak acids and a net positive surface charge predominated. The ZP and pH_PZC_ of BSBC, ZrBSBC_6.5_, ZrBSBC_12.5_, Zr–FeBSBC, Zr-FeCl_3_BSBC, Fe–BSBC and FeCl_3_–BSBC confirmed a positive in this pH range. Antimonate being in an undissociated form in the pH < pH_PZC_, an important sorption mechanism between the aqueous Sb(OH)_6_^−^ species is likely to be electrostatic attraction. The Zr content in ZrBSBC plays an important role in increasing Sb(V) sorption. However, the presence of Zr and Fe resulted in the greatest removal of Sb(V) from the solution. The presence of Zr and Fe resulted in the greatest removal of Sb(V) from the solution. This may be due to the enhanced SSA (specific surface area) from the Zr–Fe coatings on biochar surface and an increase in the positive surface charge produced compared to pristine biochar, which is responsible for higher Sb uptake. Thermodynamic results demonstrated that Sb(V) sorption was more favourable with an increase in temperature which suggested chemisorption (surface complexation) (Table 3 and Figure S9B). The empty d-orbitals on Zr and Fe might facilitate the complexation of Sb(V) through the formation of inner-sphere Zr–O–Sb, Zr–O–Fe–Sb, and Fe–O–Sb complex or via hydrogen bonding. Results from TEM and XPS demonstrate surfaced induced changes in the Sb oxidation state. TEM images suggest a concentration of Sb or potential surface precipitation on the biochars. In addition, despite the experimental systems being open to the atmosphere (i.e. oxic), XPS analysis indicates substantial surface-induced reduction of Sb(V). Biochar has previously been implicated in serving as an electron shuttle, allowing potentially for chemically induced transformation of oxidized species^31,44,49^. The presence of Sb(III) and Sb-enriched crystalline materials suggest more complex surface processes than adsorption or homogenous precipitation mechanisms in pristine, Fe or Zr modified biochars. Especially under acidic conditions, the reduction of Sb(V) to Sb(III) may indicate the surface-induced precipitation of SbO_3_. In this study, the hypothesis could not be confirmed. Nevertheless, the surface reduction of Sb(V) under oxic conditions has important implications for the application of biochars in contaminated waters or soils, due to the difference in toxicity and sorption behaviour.

**Table 3:** Thermodynamic parameters for the sorption of Sb(V) on biochars.

Biochar	∆G (kJ mol^−1^)						∆H (kJ mol^−1)^	∆S (Kl/mol.K)	R^2^	pH
	277 K	288 K	293 K	298 K	303 K	310 K				
BSBC	2.92	1.54	− 0.29	− 0.71	− 1.71	− 2.83	52.8	0.18	0.98	2
ZrBSBC_6.5_	1.58	− 0.23	− 0.48	− 1.83	− 2.57	− 3.19	43.1	0.15	0.98	2
ZrBSBC_12.5_	− 0.08	− 1.31	− 1.97	− 2.78	− 3.68	− 4.54	38.3	0.14	0.99	2
Zr–FeBSBC (1:20)	− 0.78	− 2.52	− 3.86	− 4.66	− 5.67	− 7.26	53.7	0.2	0.99	4
Zr–FeCl_3_BSBC (1:5)	− 0.36	− 1.79	− 2.48	− 3.37	− 4.67	− 5.72	45.7	0.16	0.98	4
FeBSBC	− 0.27	− 1.45	− 2.07	− 2.42	− 2.71	− 3.41	25.6	0.09	0.99	3
FeCl_3_BSBC	0.67	− 0.10	− 0.37	− 1.14	− 1.94	− 2.98	31.43	0.11	0.95	3

## Materials and methods

### Preparation of pristine biochar

Pristine biochar was produced from biosolids. The feedstock was air-dried, ground (< 1 mm, 50 mesh), and heated in a muffle furnace followed by placing in a ceramic crucible under an N_2_ atmosphere. The heating rate of 7 °C min^−1^ was employed using slow pyrolysis with holding at a peak temperature of 300 °C for 30 min^32^. The resulting biosolid biochar (BSBC) samples were cooled at room temperature inside the furnace. Afterwards, the BSBC was removed from the furnace, stored in airtight plastic containers and preserved in a desiccator for further experiments.

### Modification of biochar

The Zr–BSBC composites were synthesized by employing an *in-situ* precipitation method^49^. In this study, a solution containing 5.0 g of BSBC and 50 mL 0.1 M zirconium (IV) chloride solution (ZrOCl_2_.8H_2_O ) was brought to: i) pH 6.5 and ii) pH 12.5 through dropwise introduction of 0.1 M NaOH. The resulting suspension was aged for 12 h at room temperature. These two synthesized Zr–BSBC composites were rinsed several times by purified water to remove impurities after centrifugation at 5842 g for 15 min and followed by drying in an oven at 80 °C. The synthesized biochar composites (coded as Zr–BSBC_6.5_ and Zr–BSBC_12.5_) were preserved in a desiccator for further experiments.

Seven types of Zr–Fe and Zr–FeCl_3_ biochar composites were synthesized from Fe chips and FeCl_3_.6H_2_O at pH 6.5. The Zr to Fe molar ratios were 1:1, 1:2, 1:5, 1:10, 1:20, 1:50 and 1:100. The biochar suspensions were shaken for 24 h and centrifuged at 5842 g for 15 min, followed by decanting. Then, the synthesized biochar composites were rinsed several times by purified water, centrifuged at 5842 g for 15 min and dried in an oven at 80 °C. In addition, a Fe-only modified biochar was produced from iron chips (Fe–BSBC) and iron chloride (FeCl_3_–BSBC) as described above.

### Characterization of adsorbents

Surface charge was characterised viz. zeta potential (ZP), point of zero charge (pH_PZC_) and cation exchange capacity (CEC) using a NanoPlus HD analyser (Micromeritics, USA), Brunauer–Emmett–Teller (BET) for specific surface area (SSA). Pore size distribution and pore volume were determined using N_2_ sorption (Tristar II 3020, Micromeritics, USA) and the elemental composition (C, N, S) measured using a LECO TruMac C/N/S. The surface functional groups and morphology was investigated with Fourier transform infrared (FT-IR, Agilent Cary 600), X-ray diffraction (XRD, Empyrean, PANanalytical) and Environmental Scanning Electron Microscopy (SEM, Zeiss Sigma, Germany) equipped with a Bruker energy dispersive X-ray spectroscopy (EDS) detector. Additionally, the micromorphology of biochar samples were determined using a high-resolution transmission electron microscope (HRTEM, JEM-2100F, Japan) coupled with EDS detector (JEOL-JED-2300). Antimony in all aqueous samples was determined by using inductively coupled plasma optical emission spectrometry (ICP-OES, PerkinElmer Avio 200, USA). The elemental oxidation number, surface composition and speciation of sorbed Sb on the biochars surface also determined by XPS (ESCALAB250Xi, Thermo Scientific, UK, mono-chromated Al K alpha).

### Batch sorption experiments: pH, adsorption kinetics, and isotherms

Sorption edge investigations were achieved in the pH range of 1–10 at an initial Sb(V) concentrations of 10 mg L^−1^ with a biochar density of 4 g L^−1^ at room temperature (20 ± 2 °C). The suspension pH was controlled by addition 0.1 M HNO_3_ and/or 0.1 M NaOH. Kinetics studies were conducted using 0.1 g biochar in 25 mL solution (biochar to solution ratio = 1:250), which was added to 50 mL falcon tubes containing 5 mg/L Sb(V) for 7 d at room temperature (22 ± 0.2 °C). The background electrolyte was 0.01 M NaNO_3_ in ultrapure water. Following reactions, suspensions were centrifuged at 5842 g for 20 min and the supernatants were filtered through 0.22 µm PES filters. Kinetic data were fitted with four classical kinetic models, namely the pseudo-first-order kinetic model, pseudo-second-order kinetic model, Elovich model and Intra-particle diffusion model.

Adsorption isotherms used a similar procedure as the kinetic experiments except using a range of Sb(V) concentrations (5–250 mg L^−1^) for 72 h at pH 2–10. Four sorption isotherm models were fitted to the data, namely the Langmuir, Freundlich, Temkin and Dubinin**–**Radushkevich models (detailed information of all isotherm models in SI section).

### Influence of biochar dosage, interfering ions, ionic strength and thermodynamics

To assess the impact of adsorbent dosage on Sb(V) sorption, different dosages of biochar (solid:solution) (1:100, 1:250, 1:500 and 1:1000) were added into 50 mL centrifuge tubes maintaining pH at 2–10. Ionic strength (0.01, 0.1, 0.5 and 1.0 M of NaNO_3_), coexisting anions (Cl^−^, NO_3_^−^, SO_4_^2−^, CO_3_^2−^ and PO_4_^3−^ at concentrations of 0.01–0.1 M) and cations (0.1 M of Na, K, Mg, and Ca) were also studied. The pH of biochar samples were adjusted to 2.0 for BSBC, ZrBSBC_6.5_, ZrBSBC_12.5_, FeBSBC and 3.0 for Zr–FeBSBC (1:20), Zr–FeCl3BSBC (1:5), FeCl_3_BSBC, respectively. The Sb(V) concentration was 20 mg/L, adding 0.1 g biochar in 25 mL solution. The thermodynamic studies were conducted by varying temperatures at 4, 15, 20, 25, 30, and 37 °C. The thermodynamic parameters of the Gibbs free energy (∆G), entropy (∆S), and enthalpy (∆H) and were calculated (details are provided in SI section).

## Supplementary Information

Supplementary Information.
