# Supplementary material for: Antimonate sequestration from aqueous solution using zirconium, iron and zirconium-iron modified biochars
Source: Sci Rep. 2021 Apr 14;11:8113. doi: 10.1038/s41598-021-86978-6 (PMC8046795; doi:10.1038/s41598-021-86978-6)
Supplement: Supplementary file 1 — Supplementary Information. [file 41598_2021_86978_MOESM1_ESM.docx]

**Supporting Information for**

**Antimonate sequestration from aqueous solution using zirconium, iron and zirconium-iron modified biochars**

Md. Aminur Rahman*^a,b^, Mohammad Mahmudur Rahman^a,c^, Md Mezbaul Bahar^a,c^, Peter Sanderson^a,c^, Dane Lamb^b^*,

*^a^Global Centre for Environmental Remediation (GCER), The University of Newcastle,*

*Australia*

*b Global Innovation Centre for Advanced Nanomaterials, The University of Newcastle, Australia*

*^b^Department of Public Health Engineering (DPHE), Zonal Laboratory, Khulna-9100, Bangladesh*

***Corresponding Author**

Md. Aminur Rahman (E-mail: md.aminur.rahman@uon.edu.au)

Dane Lamb (E-mail: dane.lamb@newcastle.edu.au)

**Reagents and materials**

All chemical reagents used in this study were of analytical reagent (AR) grade from Sigma-Aldrich, NSW, Australia. The glassware and plasticware used during the experimental and analytical work were washed in 3% HNO_3_ solution followed by rinsing three times. Stock solutions of 1000 mg L^-1^ Sb(V) in Milli-Q water (resistivity of 18.3 MΩ.cm) were prepared from potassium hexahydroxyantimonate (potassium pyroantimonate, KSb(OH)_6_), Sigma-Aldrich. Zirconium (IV) oxychloride octahydrate (ZrOCl_2_.8H_2_O), iron (III) chloride hexahydrate (FeCl_3_.6H_2_O), iron chips (Fe), sodium nitrate (NaNO_3_), potassium nitrate (KNO_3_), magnesium nitrate (Mg(NO_3_)_2_), calcium nitrate (Ca(NO_3_)_2_), sodium sulphate (Na_2_SO_4_), sodium carbonate (Na_2_CO_3_), sodium phosphate monobasic (NaH_2_PO_4_), nitric acid (HNO_3_), hydrochloric acid (HCl) and sodium hydroxide (NaOH) were (Sigma-Aldrich). The biosolid biomass (BSBM) was obtained from Winmalee sewage treatment plant-Winmalee, NSW, Australia. The working feedstock of BSBM was stored at ambient temperature (approximately 24°C) after being oven dried at 80 °C for 24 h and ground to a powder (<1 mm, 50 mesh).

**Preparation of pristine biochar**

The feedstock was air-dried, ground (<1 mm, 50 mesh), and heated in a muffle furnace followed by placing in a ceramic crucible under an N_2_ atmosphere. The heating rate of 7 °C min^-1^ was employed using slow pyrolysis with holding at a peak temperature of 300 °C for 30 min ^1^. The resulting biosolid biochar (BSBC) samples were cooled at room temperature inside the furnace. Afterwards, the BSBC was removed from the furnace, stored in airtight plastic containers and preserved in a desiccator for further experiments.

**Zeta potential**

Electrophoretic mobility of biochar, often expressed as zeta potential (ZP), can be used to evaluate surface charge properties of particulate systems. ZP is the electrical potential of a sliding plane which is the interface between the stern and diffuse layers in the double layer model of colloidal particles ^2,3^. ZP was determined for each biochar sample in duplicate using a Zeta-Analyzer (NanoPlus HD, USA). All samples were dried and passed through a No. 200 sieve prior to measurement. A solution containing 0.01 g biochar in 50 mL (0.02%, w/V) 0.01 M NaNO_3_ was prepared in 50 mL centrifuge tubes. A small amount of the solution was placed into the cell. The velocity of particles moving toward a positively charged electrode is then measured to compute the ZP of each sample using Zeta-Meter. This measurement was taken 4-5 times and averaged, with duplicate samples for each biochar sample.

**Point of zero charge (PZC)**

The pH at which the net charge of a solid surface is zero, is referred to as zero point charge (PZC), which is one of the most important parameters used to describe variable-charge ^4,5^. A Zeta-Analyzer NanoPlus HD, USA was used to determine the PZC, where 0.02 g (0.02 % w/V) biochar was taken is a centrifuge tube using 0.01 M NaNO_3_ as a background electrolyte. pH (1.0-10) was adjusted by using 0.1 M HNO_3_ and/or NaOH and kept in a rotary shaker for 24 hours. After equilibrium, the samples were placed in a sonicator for 15 minutes and then biochar suspension was injected into the Zeta-analyzer. After proper calibrating the instrument, the PZC was determined from plotting the pH Vs zeta potential (where intersects the curve).

**Cation exchange capacity (CEC)**

Effects of biochars on surface charge properties were assessed by CEC and electrophoretic mobility property of biochars. Cation exchange capacity (CEC_B_) is an indicator of abundance on the surface of a material, which can be balanced by exchangeable cations ^6-8^. In this study, biochar CEC_B_ was determined by BaCl_2_ compulsive exchange modified method as described by Gillman and Sumpter (1986) ^9^. Results are inserted in **Table 1**.

**BET-N_2_ surface area**

Specific surface area (SSA) of biochar samples (<2 mm) were measured with nitrogen adsorption isotherms at liquid nitrogen temperature (-196 °C) by a Surface Area and Porosity Analyzer (Micromeritics TriStar II BET, USA). Biochar samples were degassed overnight at 60 °C under vacuum at 2 Torr before N_2_ adsorption (micromeritics VacPrep 061 Sample Degas System). The molecular surface area of 16.2 Å^2^ for N_2_ and BET (Brunauer-Emmett-Teller) ^10^ equation were used to calculate the surface area of the biochar samples. BET and Langmuir adsorption isotherms were generated to determine the single-point surface area. Pore volume and pore diameter of all biochars were also measure using a gas sorption analyzer by BET method. The mean diameter of the biochar was measured by using a Zeta plus particle size analyzer (NanoPlus HD, USA). The detection range of size of the analyzer is from 2 nm to 3 µm.

**Determination of C, N and S**

Biochars were first air-dried at 80 °C and placed into glass vials. At least two subsamples (0.15-0.20 g each) from these sample vials were measured for the contents (wt %) of C, N, and S of all biochars (<150 µm) by a CNS analyzer using LECO, USA operated in CHN mode.

**Fourier transform infrared (FT-IR)**

Fourier transform infrared (FT-IR) spectra were recorded using a Cary 600 Series (Agilent Technologies, USA) spectrometer in order to obtain the information on the nature of probable interactions between surface functional groups on the surface of biochar and antimonate ions. This was done by applying a dehydrated KBr disc technique, where biochar samples (150 µm) were mixed with spectroscopic grade KBr at a ratio 1:100 before scanning to produce sufficient transmittance. Spectra over the 4000-400 cm^-1^ range were obtained by the co-addition of 64 scans with a resolution of 4 cm^-1^ and a mirror velocity of 0.6329 cm/s.

**X-ray diffraction (XRD)**

An X-ray diffractometer (Bruker D8 Advance) was used to collect the crystallinity of the samples before and after Sb(V) sorption. All the samples were dried for 24 h at 60 °C prior to the XRD analysis. The samples were scanned from 5 to 80º by an applied current of 30 mV and a voltage of 40 kV with Co Kα radiation (1.5478 Å). The scanning rate was 1 º (2Ɵ) min^-1^ with a step size of 0.04 º. The interpretation of the XRD peaks were done using a Match3 (Crystal Impact) software (Germany).

**Scanning Electron Microscope (SEM)**

Surface morphology of the unmodified, modified and Sb-loaded biochar samples were determined under an Environmental Scanning Electron Microscope (SEM) (Zeiss Sigma, Germany) equipped with a Bruker energy dispersive X-ray spectroscopy (EDS) detector. The purpose of this study was to produce high-resolution surface morphology imaging with less electrostatic distortion. Prior to SEM imaging, all biochar samples were placed on a sample holder, following by coated with C using a high vacuum coater (Cressington 208 carbon coater, USA) for high-resolution imaging. Finally, the SEM images were taken with an accelerating voltage of 15 kV and multiple magnifications of various areas of the biochars were obtained.

**Transmission Electron Microscope (TEM)**

Microstructural analysis of biochar samples were determined by using a high-resolution transmission electron microscope (HRTEM). Before TEM analysis, the grounded biochar samples were dispersed in ethanol following by dried one drop of pristine BSBC, Sb-loaded-ZrBSBC_12.5_ and Sb-loaded-Zr-FeBSBC(1:20) suspension (after 15 minutes sonication) placed on a copper grid (Lacey carbon film, 100 µm thickness, 300 mesh, copper material). Then the samples were inserted on a sample holder and immediately placed in a JEOL-JEC-4000DS dry pumping station to ensure dry enough the samples. The high-resolution TEM images were taken by using a JEM-2100F(HR) (Japan), transmission electron microscope at an acceleration voltage of 200 kV and energy range 0-20 keV. The high-resolution elemental mapping was also taken at 5000-30000 cps and T3 mode using an energy dispersive x-ray spectroscopy (JEOL-JED-2300). TEM-overlapping mapping of Sb(V) with other elements are displayed in **Figure S11.**

**X-ray Photoelectron Spectroscopy (XPS)**

The elemental oxidation number, surface composition and speciation of sorbed Sb on the biochar surface was determined by XPS (ESCALAB250Xi, Thermo Scientific, UK, mono-chromated Al K alpa).

**pH and electrical conductivity measurement**

The pH and electrical conductivity (EC) of unmodified and all modified biochars were measured in both water and CaCl_2_ using 1:20 sample to solution ratio (w/V). pH values were measured using a pH meter (Mettler Toledo FF28, Australia), supplied with a combined electrode, calibrated using commercial pH 4.01, 7.01 and 9.23 buffers. All analysis were performed in triplicate and the average values were recorded.

**Major anions and cations analysis**

Major anion of biochar samples were also determined by ion chromatography (IC 7900, Agilent Technologies, Tokyo, Japan). Determination of Sb and other total major metals such as Na, K, Mg, Ca, Fe, Al, and P of the solid biochar were extracted by microwave digestion in aqua regia following USEPA 3051 40 method before detecting metals by using the dual view (Axial and radial) inductively coupled plasma optical-emission spectrometer (ICP-OES, PerkinElmer Avio 200, USA).

**Sorption kinetic models**

The pseudo-first-order kinetic model can be expressed using equation (S1):

| log(q_e_-q_t_) = logq_e_-[k_1_/2.303]t …………(S1) |
| --- |

Where k_1_ is the pseudo-first-order rate constant (h^-1^) and q_e_ (mg/g) is the adsorption capacity at equilibrium and q_t_ (mg g^-1^) is the adsorbed amount of Sb(V) after time t (h). Lagergren’s first-order rate constant (k_1_) and q_e_ are calculated from the slope and intercept of plots of log(q_e_-q_t_) Vs t.

The pseudo-second order model is commonly used to describe sorption kinetics in which chemical sorption controls the sorption rate and in which the number of active sorption sites on the adsorbent surface and the number of adsorbate ions in the liquid phase together determines the sorption capacity ^11^. The formation of chemical bond between adsorbate and number of adsorbing sites is the rate-limiting step ^12^. As suggested by Sheela et al. ^13^, a close agreement between theoretical and experimental adsorption capacities supports that adsorption process is well described by pseudo second order kinetic model.

The pseudo-second-order kinetics have been applied for analysing chemisorption kinetics from aqueous solutions ^14^ can be expressed using equation (S2) :

| t/q_t_ = 1/k_2_. 1/q_e_^2^ + t/q_e_ ………………..(S2) |
| --- |

Where k_2_ is the pseudo-second-order rate constant (g mg^-1^h^-1^). q_e_ and q_t_ represent the amount of Sb(V) sorbed (mg/g) at equilibrium and at any time after t (h). The parameters q_e_ and k_2_ can be calculated from slope and intercept of pseudo second order kinetics plot of t/q_t_ Vs t.

Elovic model: Zeldowitsch ^15^ developed a kinetic model which assumes that solid adsorbent surface is generically heterogeneous and no lateral interaction takes place between the adsorbed solute. Linearized form of Elovich model is represented by the following expression (S3):

| q_t_ = 1/β ln(αβ) + 1/ β (lnt) …………………….(S3) |
| --- |

where q_t_ is the amount of sorbed Sb(V) by biochar at any time t, α is the sorption kinetic at the beginning (mg/g.h) and β is the sorption constant related to the extent of surface coverage and the activation energy for chemisorption during the experiments (g/mg).The constants α and β can be obtained from the slope and intercept of the linear plot of qt Vs lnt.

The kinetic and isotherms study help to identify the sorption process, but it cannot predict the diffusion mechanism as well as rate-limiting step in the sorption process. The transfer of solute from the aqueous phase to the adsorbent particle surface may be assumed in one or more steps, e.g. film or external diffusion, intra-particle or pore diffusion, surface diffusion and adsorption on the pore surface, or a combination of more than one step ^16-18^. Generally, in a batch reactor intra-particle diffusion is often rate-limiting step. Weber and Morris ^19^ proposed an empirically found functional relationship that if intra-particle diffusion is the rate-controlling factor, uptake varies with the square root of time.

The Intra-particle diffusion model can be expressed using equation (S4) :

| q_t_ = k_id_ t^1/2^ + C ……………………..(S4) |
| --- |

where, q_t_ is the adsorption capacity at any time t and k_id_ is the intra particle diffusion rate constant (mg/g h^1/2^) and C (mg/g) is the constant which gives an impression of the film thickness of the boundary layer; indicates the larger the intercept, the greater the boundary layer effect ^20^. The slope and intercept of the linear plot of q_t_ Vs t^1/2^ was used to calculate K_id_ and C. If the plot of q_t_ Vs t^1/2^ is linear and passes through the origin, i.e. C=0, then the sorption process is considered to be only controlled by the Intraparticle diffusion ^17^ and multi-linear plot attributed to the process controlled by more than one mechanism ^21^.

**Sorption isotherm models**

The adsorbed capacity of Sb(V) concentration q (mg g^-1^), and percentage (%) of removal for all biochars were calculated according to equation S5 and S6 respectively.

q_e_ = [(C_o_-C_e_)V]/A ……………………….………………………….(S5)

% metal removal = [(C_o_-C_e_)100]/C_o_……………………...…………(S6)

where q_e_ (mg g^-1^) represents the adsorption capacity of Sb(V) onto the biochars at equilibrium conditions, C_o_ and C_e_ refer to the original and final (equilibrium) Sb(V) concentrations in solution (mg L^-1^), respectively, V is the total volume (L) of the Sb(V) solution and A (g) indicates the oven dried amount of biochar.

Four sorption isotherm models were used to fit the equilibrium experimental data of Sb(V) sorption to unmodified and modified biochars, namely the Langmuir, Freundlich, Temkin and Dubinin-Radushkevich models **(Table 4)**. The Langmuir isotherm assumes that the valid monolayer sorbent homogenously covering the sorbent surface consists of finite identical binding sites with equal sorption activated energy and none of the binding sites of adsorbent affects its neighbours ^22,23^ thus adopts uniform energies of sorption on the surface and no transmigration sorbate in the plane of the surface whereas the Freundlich isotherm is based on the assumption that multilayer sorption on an energetically heterogeneous surface and can be used to describe the chemisorption process.

The equation (S7) is used to describe in the Langmuir model (monolayer model) ^24^:

| Q_e_ = Q_m_ C_e_ K_L_/(1 + C_e_K_L_) …………………………………..(S7) |
| --- |

Where Q_e_ is the equilibrium adsorbed concentration of Sb(V) in solid phase (mg g^-1^), Q_m_ is the maximum sorption capacity (mg g^-1^) of Sb(V), represents complete monolayer coverage of sorbent with sorbate.C_e_ (mg L^-1^) is the equilibrium Sb(V) concentration and K_L_ is an equilibrium constant (L mg^-1^) related to binding strength. The constants are calculated from slope and intercept of the linear plots of C_e_/q_e_ versus C_e_, respectively. Additionally, the important characteristics of the Langmuir isotherm can be explained by separation factor R_L_, which is calculated by the equation (S8).

| R_L_= 1/1+K_L_C_0_ …………………………………………. | (S8) |
| --- | --- |

Where C_0_ (mg/L) is the initial concentration of Sb(V). There are four probabilities for the R_L_ value; (i) for favourable sorption, 0<R_L_<1, (ii) for unfavourable sorption, R_L_>1, (iii) for linear sorption, R_L_ = 1, and (iv) for irreversible sorption, R_L_ = 0.

The Freundlich model assumes a heterogeneous adsorption surface and active sites with different energy ^23^. The Freundlich equation (nonlinear model) is an empirical adsorption model usually expressed as the following equation (S9) ^25^.

| Q_e_ = K_F_ C_e_^1/n^ ……………………..…(S9) |
| --- |

Where Q_e_ and C_e_are defined previously, K_F_ is a Freundlich affinity coefficient related to the measure of the adsorption capacity and n is a Freundlich exponential coefficient measure of the adsorption intensity. Linear form of Freundlich equation is expressen by equation (S10):

| logq_e_ = 1/n logC_e_ + logK_f_ …………………(S10) |
| --- |

Temkin isotherm model considered the effects of some indirect sorbate/adsorbate interactions on adsorption isotherms and suggested that, the heat of adsorption of all the molecules in the layer would decrease linearly because of increase with surface coverage. The Temkin isotherm assumes linear rather than logarithm decrease of heat of adsorption while ignoring extremely low and very high concentration. It also assumes uniform distribution of bounding energy up to some maximum bonding energy. The Temkin isotherm has been generally applied in the following equation (S11):

| Qe = BlnA + BlnCe ……………………..(S11) |
| --- |

Where q_e_ is the amount of adsorbate adsorbed at equilibrium (mg/g); C_e_ is the concentration of adsorbate in solution at equilibrium (mg/L). B is a constant related to the heat of adsorption and it is defined by the expression B= RT/b, b is the Temkin constant (J/mol), T is the absolute temperature (K), R is the gas constsnt (8.314 J/mol K), and A is the Temkin isotherm constant (L/g). From the plot of qe Vs lnCe, B and A can be calculated from the slopes (B) and intercepts (BlnA) respectively.

The classical derivation of the Temkin isotherm deals with structural heterogeneity and assumes a constant distribution of sites in a range of energies extending from a highest X down to zero. It has also been suggested that the Temkin isotherm properly describes the case of induced heterogeneity among the adparticles during the adsorption process.

The Dubinin-Radushkevich (D-R) isotherm model is another empirical model, which initially formulated for the sorption process following a pore filling mechanism, gives insight into the biomass porosity as well as the adsorption energy. The D-R isotherm model can be used to determine whether the nature of the adsorption process is physical or chemical. It is generally applied to express the sorption process occurred onto homogeneous and heterogeneous surfaces. A distinguishing feature of the D-R isotherm is the fact that it is temperature dependent; hence, when sorption data at different temperatures are plotted as a function of logarithm of amount sorbed versus the square of potential energy, all suitable data can be determined. The non-linear expression of Dubinin-Radushkevich isotherm model can be illustrated by equations S12-S13

| lnq_e_ = lnq_m_ - βɛ^2^ ………………(S12) |
| --- |

where q_e_ is the amount of metal ions sorbed per unit weight of adsorbent (mg/L); q_m_is the maximum adsorption capacity (mg/g); β is the activity coefficient related to the mean free energy of adsorption (D-R constant), E (mol^2^/J^2^); and ɛ is the Polanyi potential can be expressed as follows:

| ɛ = RTln(1 + 1/C_e_) ……………..(S13) |
| --- |

The mean free energy of activation (E; kJ/mol) is expressed by equation (S14)

| E=1/√2β …………………..(S14) |
| --- |

Where R is the gas constant (8.314 J/mol K) and T is the temperature (K). q_m_ and β (mol^2^/kJ^2^) can respectively be calculated from the intercept and the slopes of the plot of lnq_e_ Vs ɛ^2^.

**Thermodynamic studies**

The change of entropy (∆S) and enthalpy (∆H) are important thermodynamic parameters for the identification of spontaneous process. The thermodynamic parameters ∆S, ∆H and the Gibbs free energy (∆G) were calculated using the following equations (S15-17) ^22,26^:

∆G = -RTlnK ……………………………….(S15)

Kc = q_e_/C_e_ ………………………………(S16)

∆G = ∆H – T∆S ……………………………….(S17)

Where R is the ideal gas law constant [8.314×10^-3^kJ/(mol.K), T is the absolute temperature (K); and Kc is the distribution coefficient, which is the ratio of the equilibrium sorption quantity to the equilibrium concentration of Sb(V).

The final equation can be written as:

lnK = ∆S/R – ∆H/(RT) ………………………..(S18)

Based on equation (S16), ∆H and ∆S parameters can be calculated from the slope and intercept, respectively, of the plot of lnKc versus 1/T such as using equation (S19):

∆H = – Slope × R and ∆S = Intercept × R ………………….(S19)

**Effect of pH on sorption**

Sb(V)) exists in aqueous solution as oxyanion or neutral species and are controlled by the following acid dissociation equilibrium reactions ^27^.

| Sb(OH)_5_ | + | H_2_O | → | Sb(OH)_6_^-^ | + H^+^ ………........ | pKa | =2.72 |
| --- | --- | --- | --- | --- | --- | --- | --- |

It is apparent that the dissociation depends on the acidity of the solution. In acidic pH range, the proportion of Sb(OH)_6_^-^ increases with an increase of pH. At lower pH, higher sorption of Sb(V) may be due to the electrostatic interaction of positively charged adsorbent sites and the negatively charged Sb(OH)_6_^-^, and HSb_2_S_4_^-^ species. At most acidic conditions (pH<5), Sb(V) is known to exist in the negative ionic form ^28^. Moreover, the formation of oligomers such as Sb_12_(OH)_64_^4-^ is favoured for Sb(V) on aqueous solutions at pH values <7 ^29^. Thus under acidic conditions, across-surface Sb(III) oligomerization is likely to stabilize the sorbed Sb(V). Correspondingly, at below pH 5, the biochar composites should behave as weak acids and form positive surface sites for anionic Sb(V) sorption by electrostatic interaction. At low pH, the positively charged ZrO, Zr-O-Fe, Fe-O coated sorbents; sorbed Sb(OH)_6_^-^, and HSb_2_S_4_^-^ species due to -OH, -COOH groups are protonated, which has a high concentration of these functional groups leads to a positively charged surface. Therefore, at the lower pH (pH 1-3), unmodified and modified biochar materials indicate that this is more efficient for Sb(V) sorption ^30^. This observation might be due to some buffering phenomenon that occurs during sorption reaction.

| **Table S1.** Zeta potential of pristine and modified biochars | | | | | | | |
| --- | --- | --- | --- | --- | --- | --- | --- |
| pH | **Zeta potential (mV)** | | | | | | |
|  | BSBC | ZrBSBC_6.5_ | ZrBSBC_12.5_ | Zr-FeBSBC (1:20) | Zr-FeCl_3_BSBC (1:5) | FeBSBC | FeCl_3_BSBC |
| 2 | +5.21 | +8.87 | +17.37 | +19.87 | +25.02 | +22.4 | +12.23 |
| 3 | +2.63 | +7.77 | +21.91 | +25.75 | +21.15 | +7.84 | +16.2 |
| 4 | -2.03 | -3.48 | -5.12 | +6.83 | +15.43 | -2.9 | -4.66 |
| 5 | -7.65 | -4.25 | -13.32 | +3.87 | +9.98 | -5.33 | -7.46 |
| 6 | -10.28 | -8.4 | -20.28 | +2.24 | -4.78 | -6.87 | -6.66 |
| 7 | -13.54 | -10.58 | -31.26 | -7.51 | -13.79 | -15.93 | -6.86 |
| 8 | -14.73 | -13.53 | -35.54 | -17.75 | -12.21 | -6.91 | -7.05 |
| 9 | -17.36 | -18.21 | -27.28 | -22.34 | -16.45 | -7.23 | -10.13 |
| 10 | -20.59 | -21.11 | -25.87 | -25.12 | -19.72 | -11.57 | -12.57 |
| 11 | -24.21 | -23.25 | -31.55 | -28.57 | -25.68 | -19.81 | -15.36 |

| **Table S2**. Physico-chemical characteristics of biochars | | | | | | | | | |
| --- | --- | --- | --- | --- | --- | --- | --- | --- | --- |
| Biochar | Specific surface area (BET), (m^2^/g) | Pore  volume  (cm^3^ g^-1^) | Pore size/diameter (nm) | Average particle size (nm) | pH_PZC_ | EC  (mS/cm) | pH | | CEC  (cmol(+)/kg) |
|  |  |  |  |  |  |  | In  H_2_O | In  CaCl_2_ |  |
| BSBC | 4.64 | 0.006 | 6.51 | 1292 | 3.6 | 0.32 | 7.12 | 5.98 | 8.1 |
| ZrBSBC_6.5_ | 35.78 | 0.033 | 4.01 | 167 | 3.7 | 12.51 | 6.78 | 6.13 | 7.62 |
| ZrBSBC_12.5_ | 75.85 | 0.06 | 3.37 | 79 | 3.8 | 10.34 | 8.97 | 8.37 | 6.24 |
| Zr-FeBSBC(1:20) | 27.87 | 0.038 | 6.22 | 215 | 6.2 | 22.64 | 4.48 | 4.19 | 6.11 |
| Zr-FeCl_3_BSBC(1:5) | 25.51 | 0.019 | 3.92 | 235 | 5.7 | 18.57 | 5.64 | 5.17 | 5.63 |
| FeBSBC | 6.60 | 0.007 | 5.75 | 909 | 3.9 | 18.96 | 5.41 | 5.28 | 5.11 |
| FeCl_3_BSBC | 24.02 | 0.027 | 4.80 | 249 | 3.8 | 20.54 | 5.88 | 5.45 | 5.06 |

| **Table S3.** Total elemental composition of biochars | | | | | | | | | | | |
| --- | --- | --- | --- | --- | --- | --- | --- | --- | --- | --- | --- |
| Biochar | Mass % | | | | mg/g | | | | | |  |
|  | C | N | S | Zr | Na | K | Mg | Ca | Fe | Al | P |
| BSBC | 22.88 | 3.60 | 1.12 | - | 1.99 | 4.24 | 7.76 | 22.97 | 100.69 | 29.65 | 54.86 |
| ZrBSBC_6.5_ | 21.93 | 3.32 | 1.56 | 6.9 | 7.86 | 2.68 | 6.69 | 18.26 | 83.69 | 21.81 | 2.16 |
| ZrBSBC_12.5_ | 20.71 | 3.10 | 1.11 | 9.7 | 26.99 | 2.83 | 8.82 | 18.84 | 79.12 | 20.37 | 0.83 |
| Zr-FeBSBC (1:20) | 18.73 | 2.38 | 0.64 | 4.3 | 34.87 | 1.37 | 0.83 | 1.81 | 270.1 | 10.62 | 4.66 |
| Zr-FeCl_3_BSBC (1:5) | 17.88 | 2.76 | 0.85 | 3.1 | 27.18 | 1.59 | 4.35 | 11.88 | 238.82 | 17.32 | 6.12 |
| FeBSBC | 21.83 | 3.27 | 1.05 | - | 71.54 | 1.82 | 4.19 | 12.37 | 176.21 | 19.26 | 43.47 |
| FeCl_3_BSBC | 22.96 | 3.47 | 1.67 | - | 12.75 | 2.34 | 4.05 | 15.04 | 228.17 | 22.10 | 42.86 |

| 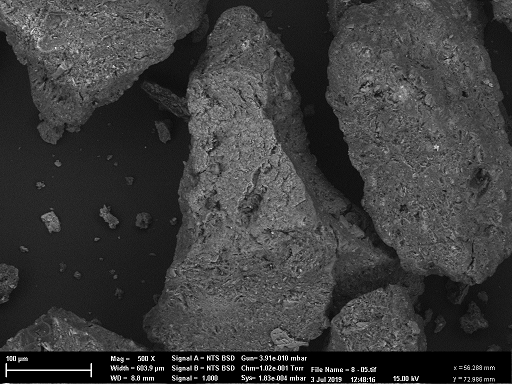 | 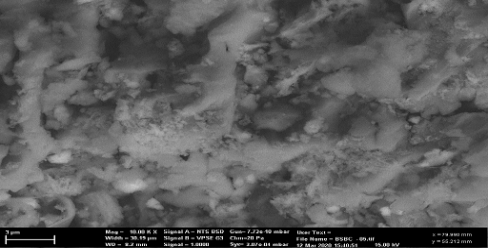 | |  |
| --- | --- | --- | --- |
| A(i) BSBC | A(ii) BSBC | | A(iii) Sb loaded BSBC |
|  |  | |  |
| B(i) ZrBSBC_6.5_ | B(ii) ZrBSBC_6.5_ | | B(iii) Sb loaded ZrBSBC_6.5_ |
|  |  | |  |
| C(i) ZrBSBC_12.5_ | C(ii) ZrBSBC_12.5_ | | C(iii) Sb loaded ZrBSBC_12.5_ |
|  |  | |  |
| D(i) Zr-FeBSBC (1:20) | D(ii) Zr-FeBSBC (1:20) | | D(iii) Sb loaded Zr-FeBSBC(1:20) |
|  | |  |  |
| E(i) Zr-FeCl_3_BSBC (1:5) | | E(ii) Zr-FeCl_3_BSBC (1:5) | E(iii) Sb loaded Zr-FeCl3BSBC(1:5) |
|  | |  |  |
| F(i) FeBSBC (10 µm) | | F(ii) FeBSBC (1 µm) | F(iii) Sb loaded FeBSBC |
|  | |  |  |
| G(i) FeCl_3_BSBC | | G(ii) FeCl_3_BSBC | G(iii) Sb loaded FeCl3BSBC |
| **Figure S1.** SEM images of biochars before A(i-ii) to G(i-ii), and after Sb(V) sorption A(iii)-G(iii) | | | |

| **Counts** | 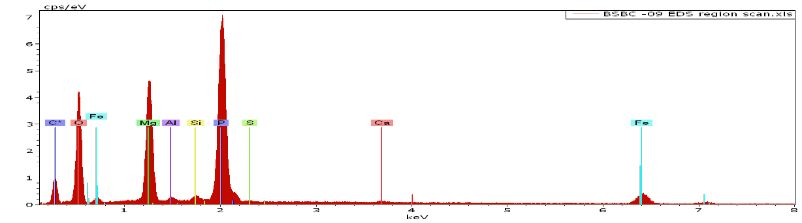  A(i) BSBC | 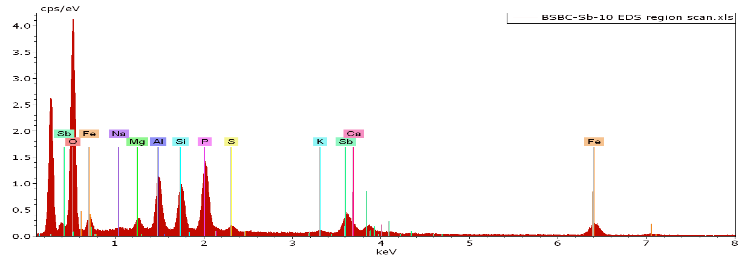  A(ii) BSBC + Sb |
| --- | --- | --- |
|  | 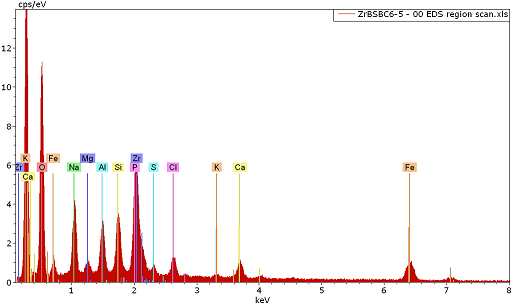  B(i) ZrBSBC_6.5_ | 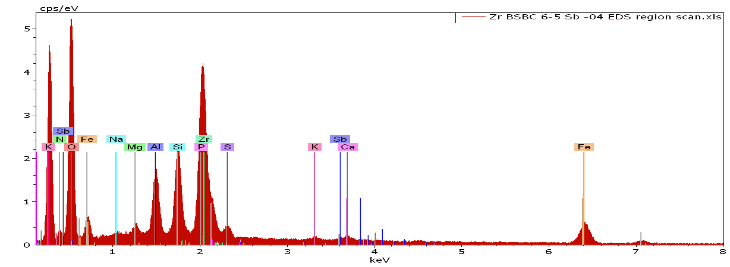  B(ii) ZrBSBC_6.5_ + Sb |
|  | 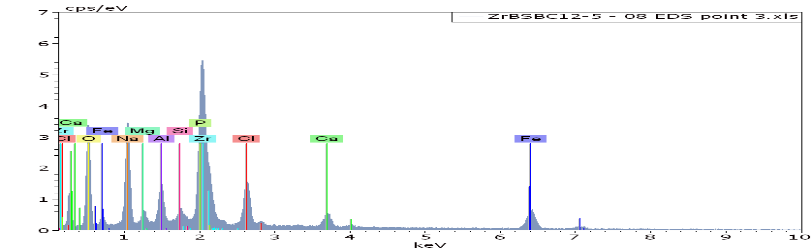  C(i) ZrBSBC_12.5_ | 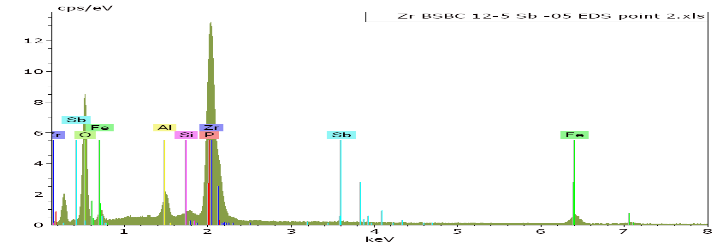  C(ii) ZrBSBC_12.5_ + Sb |
|  | 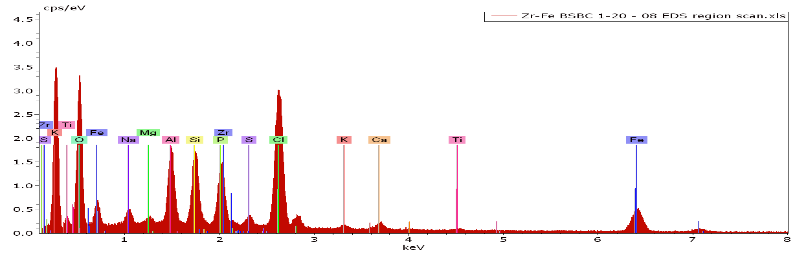  D(i) Zr-FeBSBC(1:20) | 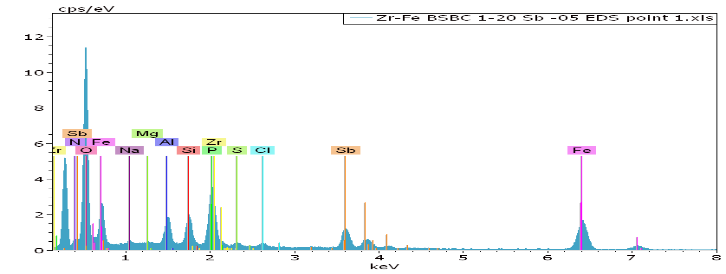 |
|  | 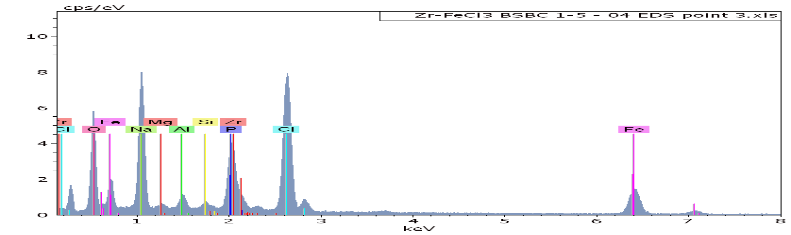  E(i) Zr-FeCl_3_BSBC(1:5) | 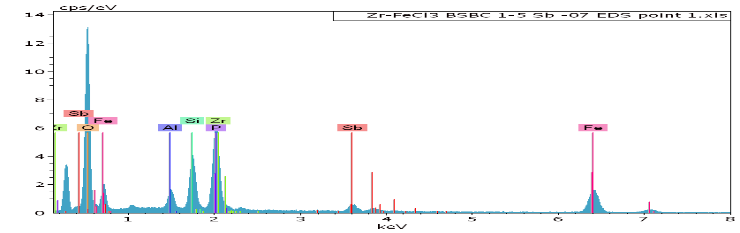  E(ii) Zr-FeCl_3_BSBC(1:5) + Sb |
|  | 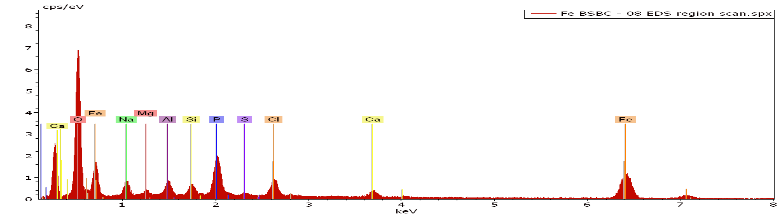  F(i) FeBSBC | 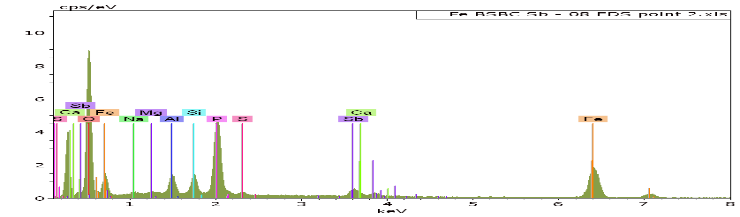  F(ii) FeBSBC + Sb |
|  | 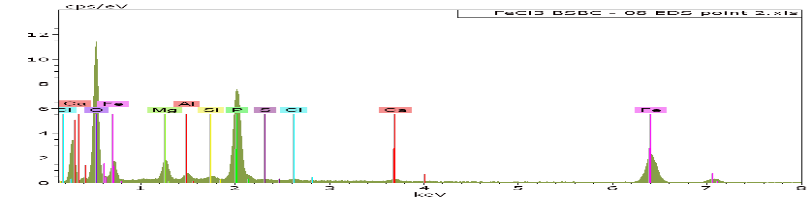  G(i) FeCl_3_BSBC | 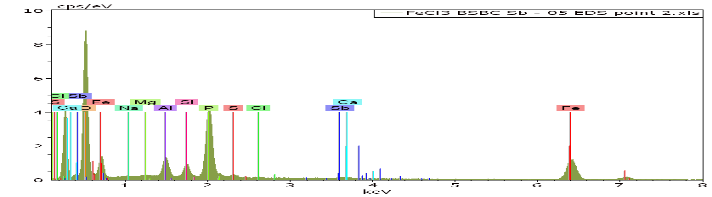  G(ii) FeCl_3_BSBC + Sb |
|  | **keV** | |
|  | **Figure S2.** EDS spectra of biochars A(i)-G(i) before, and A(ii)-G(ii) after, Sb(V) sorption with SEM | |

| **(A) BSBC** | **O** | **P** | **Ca** | **Al** |
| --- | --- | --- | --- | --- |
| 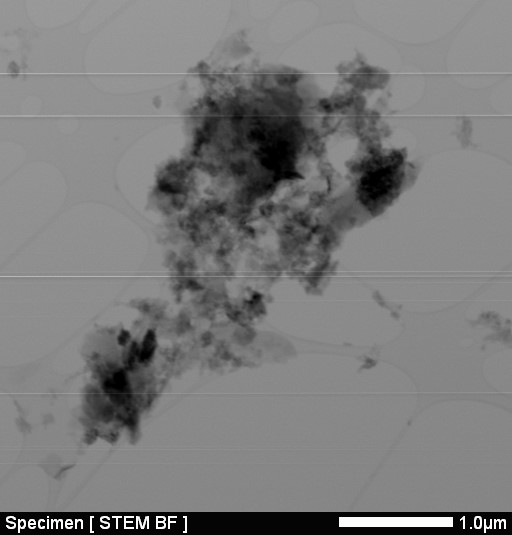 | 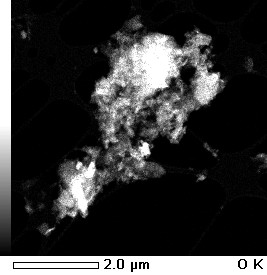 | 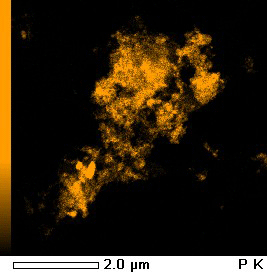 | 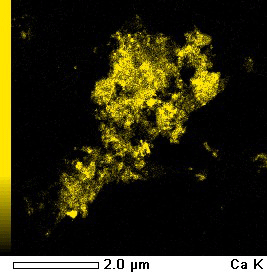 | 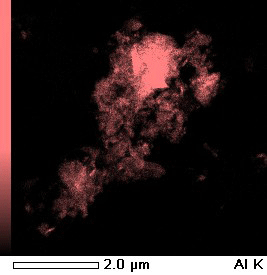 |
|  |  | **Si** | **S** | **Fe** |
|  |  | 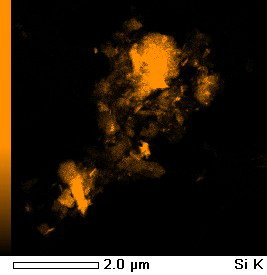 | 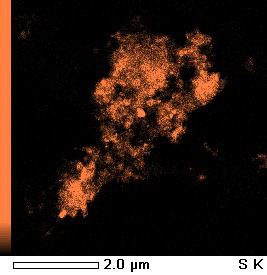 | 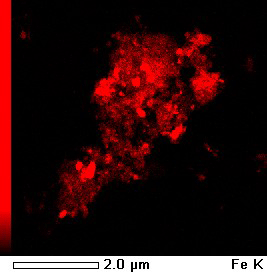 |
| **(B) BSBC + Sb** | **O** | **P** | **Ca** | **Al** |
| 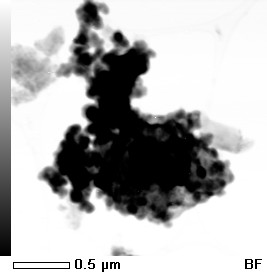 | 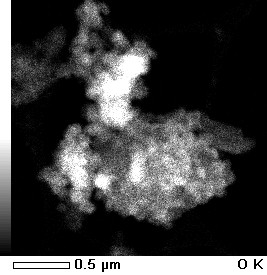 | 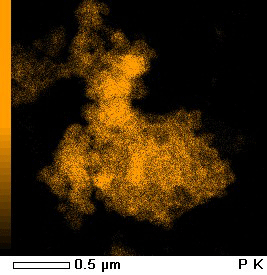 | 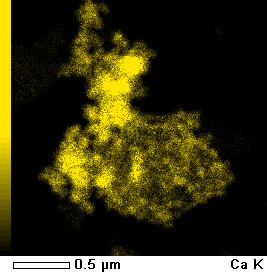 | 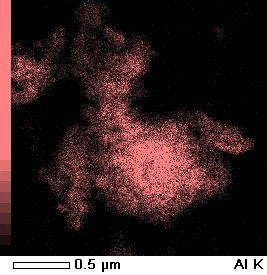 |
|  | **Sb** | **Si** | **S** | **Fe** |
|  | 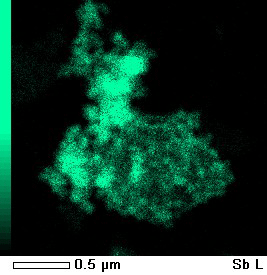 | 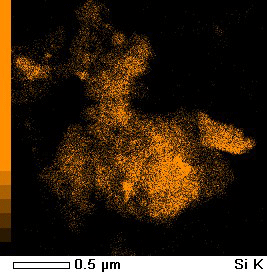 | 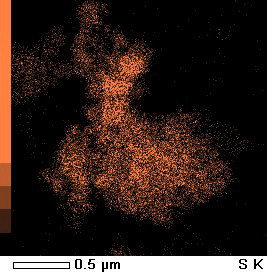 | 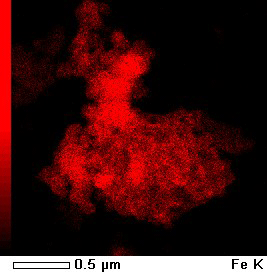 |
| **(C) ZrBSBC_12.5_ + Sb** | **O** | **P** | **Ca** | **Al** |
| 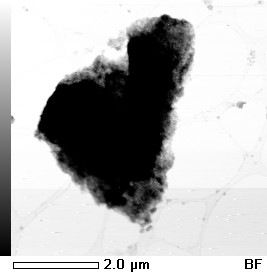 | 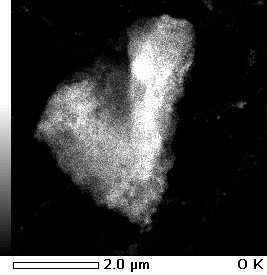 | 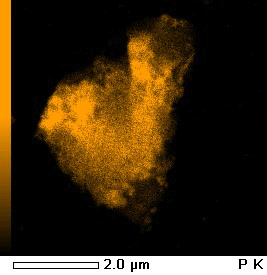 | 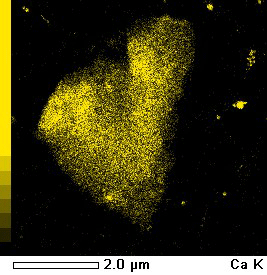 | 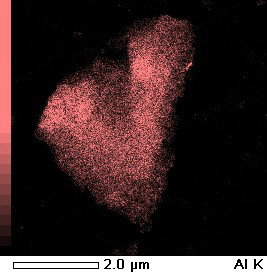 |
| **Zr** | **Sb** | **Si** | **S** | **Fe** |
| 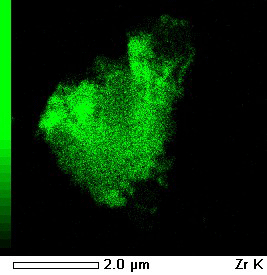 | 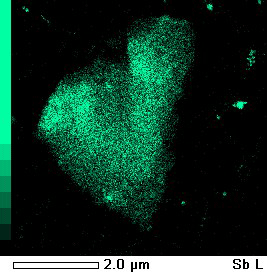 | 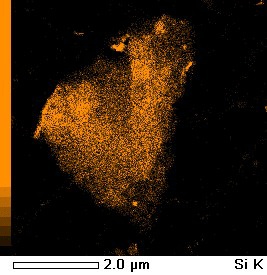 | 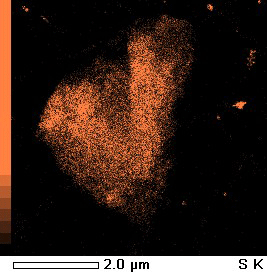 | 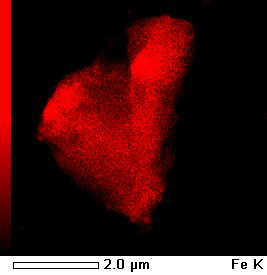 |
| **(D) Zr-FeBSBC(1:20) + Sb** | **O** | **P** | **Ca** | **Al** |
| 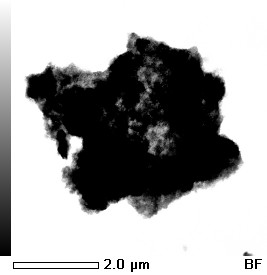 | 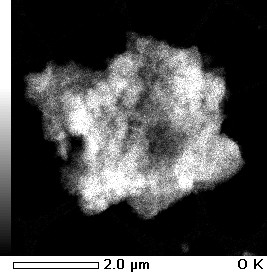 | 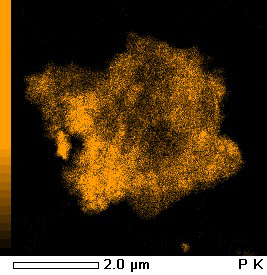 | 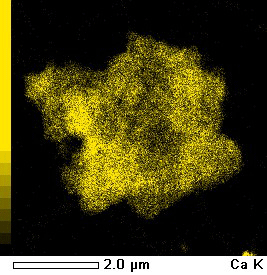 | 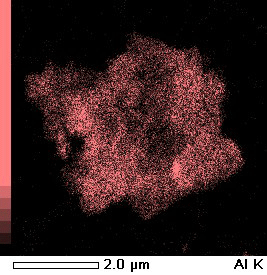 |
| **Zr** | **Sb** | Si | **S** | **Fe** |
| 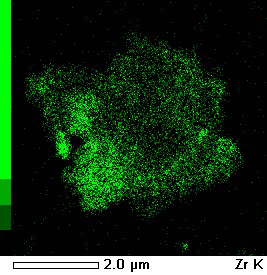 | 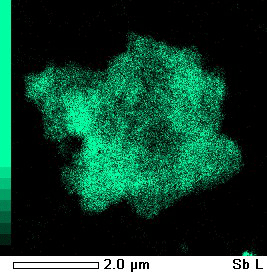 | 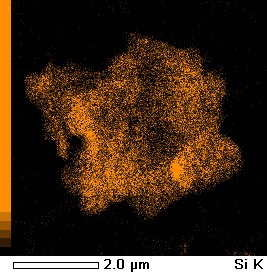 | 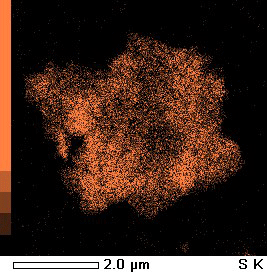 | 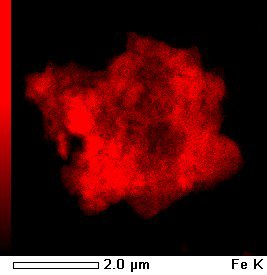 |
| **Figure S3.** TEM elemental images of (A) Pristine BSBC, (B) Sb-loaded BSBC, (C) Sb-loaded ZrBSBC_12.5_, and (D) Sb-loaded Zr-FeBSBC(1:20) | | | | |

|   A (BSBC) |   B (BSBC + Sb) |
| --- | --- |
|   C (Zr-BSBC_12.5_) |   D (Zr-BSBC_12.5_ + Sb) |
|   E (Zr-FeBSBC(1:20) |   F (Zr-FeBSBC(1:20) + Sb) |
| **Figure S4.** Raw TEM-EDS of (A) BSBC, (B) BSBC + Sb, (C) Zr-BSBC_12.5_, (D) Zr-BSBC_12.5_ + Sb, (E) Zr-FeBSBC(1:20) and (F) Zr-FeBSBC(1:20) + Sb; using Cu grid | |

| **Intensity (CPS)** | 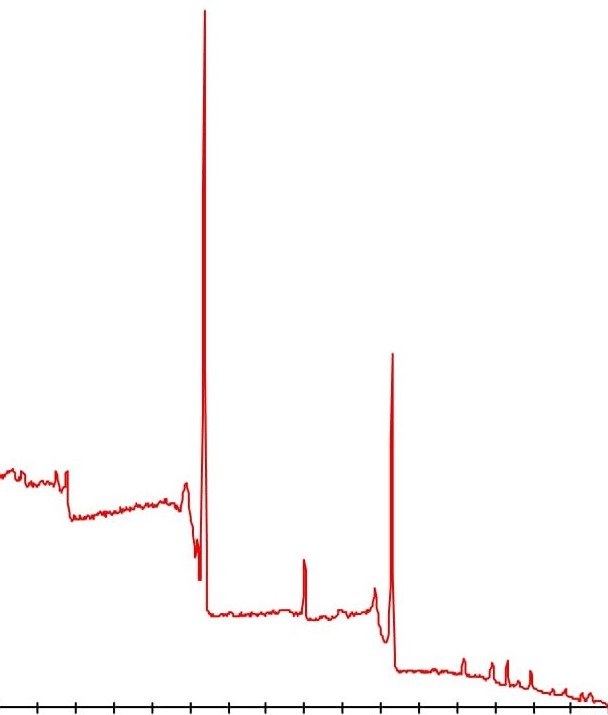  Fe 2p3  P 2p  C 1s  N 1s  O 1s + -Sb 3d5  **(A)** | | | | | | | | |
| --- | --- | --- | --- | --- | --- | --- | --- | --- | --- |
|  | 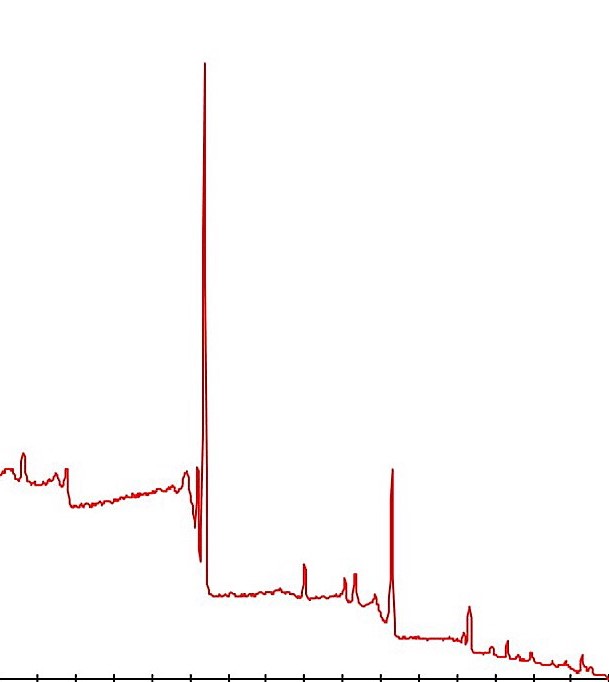  Zr 3d  O 1s + -Sb 3d5  P 2p  C 1s  N 1s  Fe 2p3 | | | | | | | | |
|  | 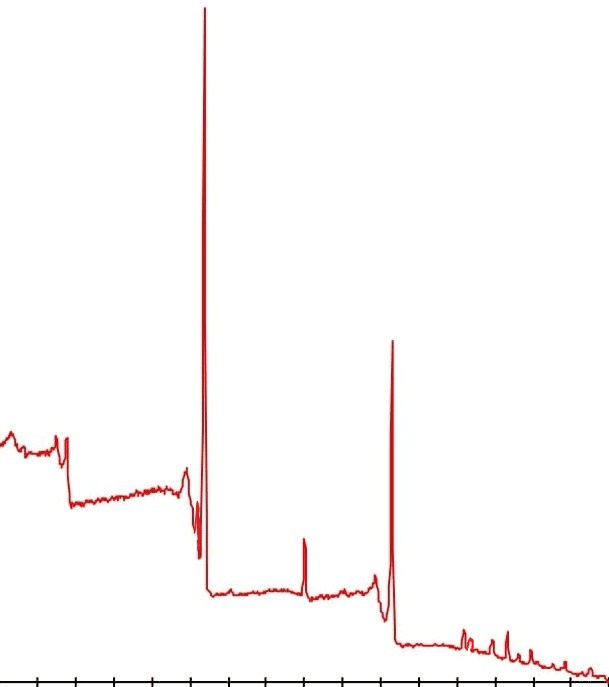  O 1s + -Sb 3d5  Fe 2p3  N 1s  C 1s  Zr 3d  P 2p  **(B)**  **(C)** | | | | | | | | |
|  | 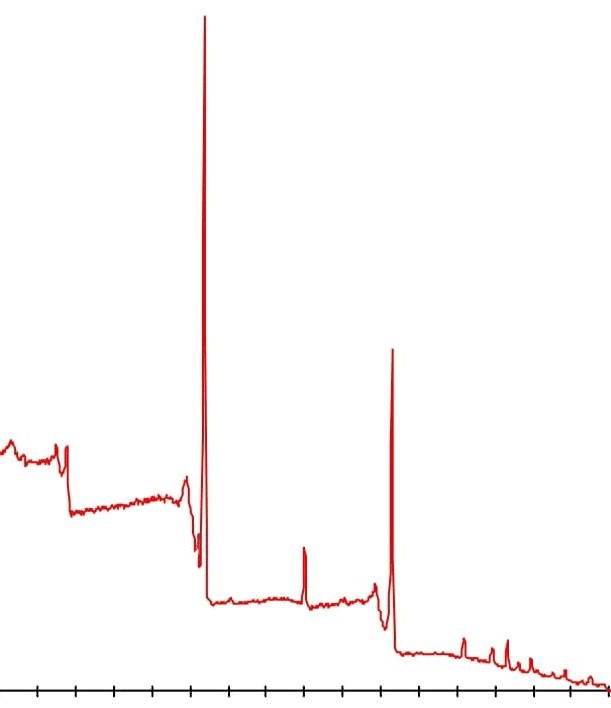  P 2p  C 1s  N 1s  O 1s + -Sb 3d5  Fe 2p3  **(D)** | | | | | | | | |
|  | 0 | 100 | 200 | 300 | 400 | 500 | 600 | 700 |  |
|  | **Binding Energy (eV)** | | | | | | | | |
|  |  | | | | | | | | |
| **Figure S5.** XPS survey profile of Sb-loaded (A) BSBC, (B) ZrBSBC_12.5_, (C) Zr-FeBSBC(1:20) and (D) FeBSBC | | | | | | | | | |

|  |
| --- |
| **Figure S6. P**oint of zero charge (PZC) of biochars |

| **(A)** | **(B)** |
| --- | --- |
| **(C)** | **(D)** |
| **Figure S7.** Kinetic models: First order (A), Second order (B), Elovich (C), and Intraparticle diffusion model (D) (Initial Sb(V) concentration was 5 mg/L, biochar density was 4 g/L at 22 ºC) | |

| **% removal, Sb(V)** | **(A)** | **(B)** |
| --- | --- | --- |
|  | **Anions** | **Cations** |
|  | **Figure S8.** Influence of major anions (A), and major cations (B), on Sb(V) sorption (Initial Sb concentration was 20 mg/L, biochar density was 4 g/L, at 22 °C) | |

| **Sorbed Sb(V), mg/g** | **(A)** | **(B)** |
| --- | --- | --- |
|  | **NaNO_3_ (M)** | **1/T** |
|  | **Figure S9.** Influence of ionic strength (A), and temperature (B), on Sb(V) sorption (Initial concentration 20 mg/L, biochar dosage 4 g/L, at 22 °C). | |

| **Intensity** | 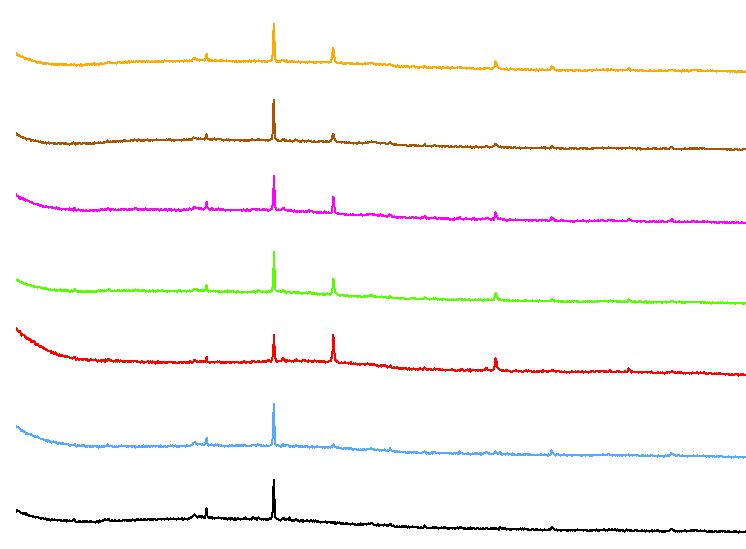  **(A)**  ZrBSBC_6.5_  BSBC  Zr-FeBSBC(1:20)  FeCl_3_BSBC  FeBSBC  Zr-FeCl_3_BSBC(1:5)  ZrBSBC_12.5_ | | | | | | | |
| --- | --- | --- | --- | --- | --- | --- | --- | --- |
|  | 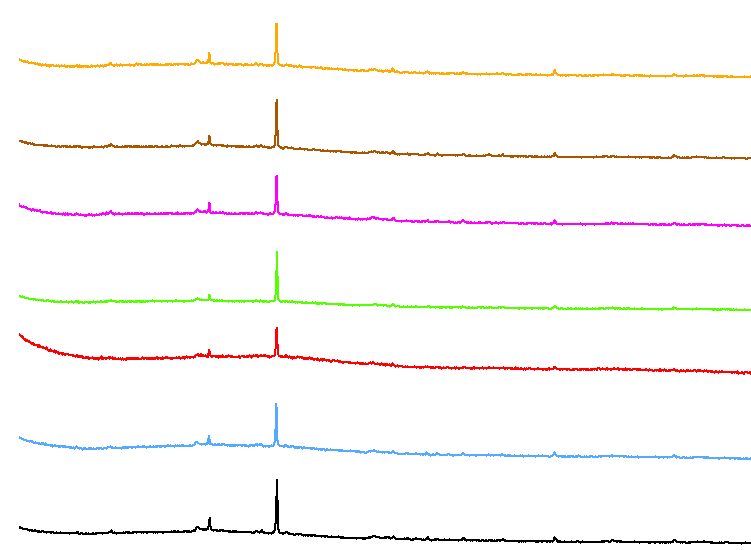  **(B)**  BSBC + Sb  ZrBSBC_6.5_ + Sb  ZrBSBC_12.5_ + Sb  Zr-FeBSBC(1:20) + Sb  Zr-FeCl_3_BSBC(1:5) + Sb  FeBSBC + Sb  FeCl_3_BSBC + Sb | | | | | | | |
|  | 10 | 20 | 30 | 40 | 50 | 60 | 70 | 80 |
|  | **2º (Theta)** | | | | | | | |
|  | **Figure S10.** X-ray diffraction (XRD) pattern of biochars before (A), and after (B), Sb(V) sorption | | | | | | | |

| (A) BSBC + Sb | Fe | P | Sb | O | Overlay (Fe P Sb O) |
| --- | --- | --- | --- | --- | --- |
| 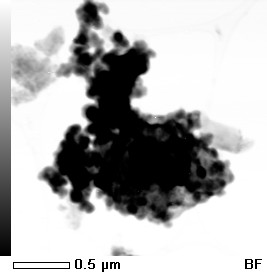 | 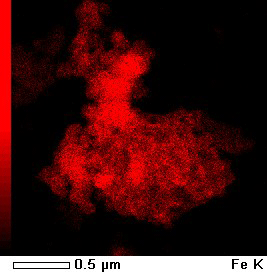 | 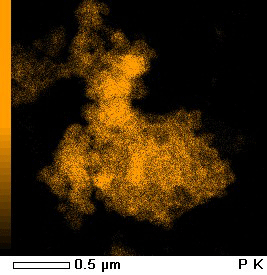 | 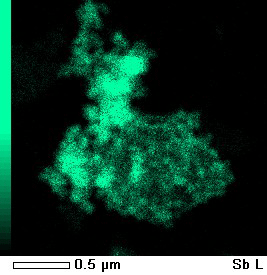 | 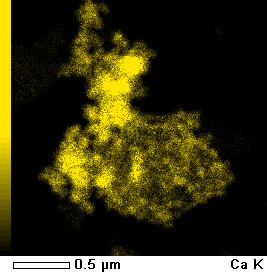 | 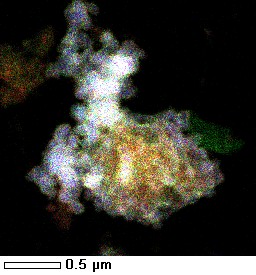 |
| (B) Zr-BSBC_12.5_ + Sb | S | Zr | Sb | Ca | Overlay (S Zr Sb Ca) |
| 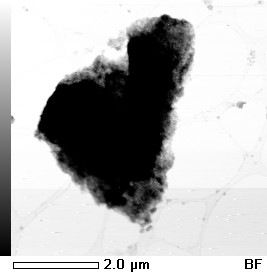 | 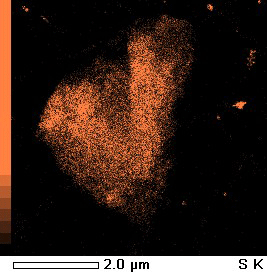 | 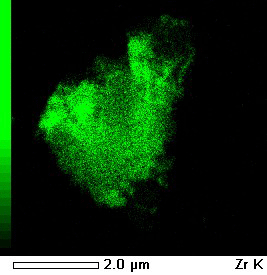 | 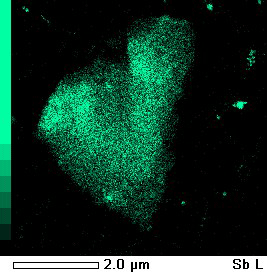 | 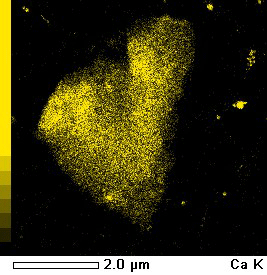 | 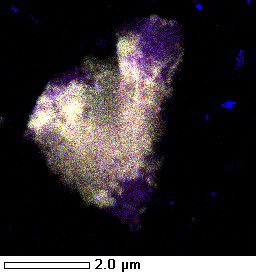 |
| (C) Zr-FeBSBC + Sb | P | Zr | Sb | Fe | Overlay (P Zr Sb Fe) |
| 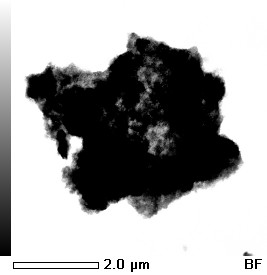 | 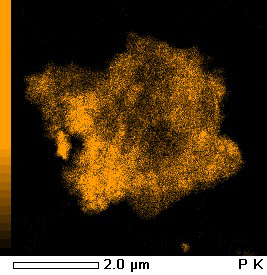 | 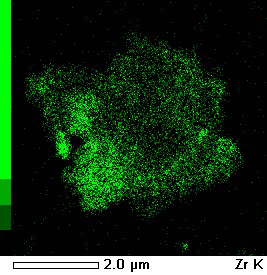 | 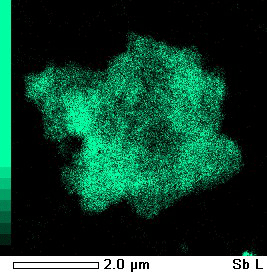 | 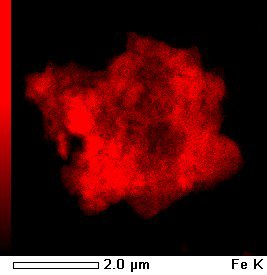 | 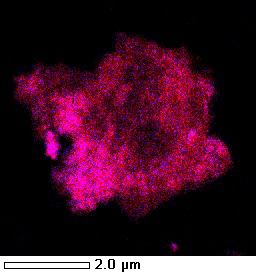 |
| **Figure S11.** Overlay mapping of Sb(V) with other elements onto (A) BSBC, (B) ZrBSBC12.5, and (C) Zr-FeCl3BSBC by TEM-EDS | | | | | |

|  |
| --- |
| **Figure S12.** Separation factor as a function of initial concentration of Sb(V) |

| **(A)** |
| --- |
| **Biochar dose, g/L** |
| **Figure S13.** Effect of biochar dosages (Initial concentration was 20 mg/L at 22 °C) (Initial concentration was 20 mg/L, biochar density was 4 g/L, at 22 °C) on Sb(V) sorption. |

| **% Sb(V) desorption** | 1. **BSBC** | **(D) Zr-FeCl_3_BSBC (1:5)** |
| --- | --- | --- |
|  | **(B) ZrBSBC_12.5_** | **(E) FeBSBC** |
|  | **(C) Zr-FeBSBC(1:20)** | **(F) FeCl_3_BSBC** |
|  | **Desorbing agents** | |
|  | **Figure S14**. Desorption of Sb(V) from (A) BSBC, (B) ZrBSBC_12.5_, (C) Zr-FeBSBC(1:5), (D) Zr-FeCl_3_BSBC (1:5), E) FeBSBC and (F) FeCl_3_BSBC (Biochar density was 4 g/L under optimum conditions) | |

**References**

1 Rahman, M. A. *et al.* Removal of arsenate from contaminated waters by novel zirconium and zirconium-iron modified biochar. *Journal of Hazardous Materials*, 124488 (2020).

2 Appel, C., Ma, L. Q., Rhue, R. D. & Kennelley, E. Point of zero charge determination in soils and minerals via traditional methods and detection of electroacoustic mobility. *Geoderma* **113**, 77-93 (2003).

3 Jiang, S. *et al.* Characterization of hard-and softwood biochars pyrolyzed at high temperature. *Environmental geochemistry and health* **39**, 403-415 (2017).

4 Morais, F. I., Page, A. & Lund, L. The Effect of pH, Salt Concentration, and Nature of Electrolytes on the Charge Characteristics of Brazilian Tropical Soils 1. *Soil Science Society of America Journal* **40**, 521-527 (1976).

5 Parks, G. A. & Bruyn, P. D. The zero point of charge of oxides1. *The Journal of Physical Chemistry* **66**, 967-973 (1962).

6 Jiang, J., Xu, R.-k., Jiang, T.-y. & Li, Z. Immobilization of Cu (II), Pb (II) and Cd (II) by the addition of rice straw derived biochar to a simulated polluted Ultisol. *Journal of hazardous materials* **229**, 145-150 (2012).

7 Mukherjee, A., Zimmerman, A. & Harris, W. Surface chemistry variations among a series of laboratory-produced biochars. *Geoderma* **163**, 247-255 (2011).

8 Zhao, L., Cao, X., Zheng, W., Wang, Q. & Yang, F. Endogenous minerals have influences on surface electrochemistry and ion exchange properties of biochar. *Chemosphere* **136**, 133-139 (2015).

9 Gillman, G. & Sumpter, E. Modification to the compulsive exchange method for measuring exchange characteristics of soils. *Soil Research* **24**, 61-66, doi:<http://dx.doi.org/10.1071/SR9860061> (1986).

10 Brunauer, S., Emmett, P. H. & Teller, E. Adsorption of gases in multimolecular layers. *Journal of the American chemical society* **60**, 309-319 (1938).

11 Liu, K. *et al.* Understanding the adsorption of PFOA on MIL-101 (Cr)-based anionic-exchange metal–organic frameworks: comparing DFT calculations with aqueous sorption experiments. *Environmental science & technology* **49**, 8657-8665 (2015).

12 Ho, Y.-S. & McKay, G. Pseudo-second order model for sorption processes. *Process biochemistry* **34**, 451-465 (1999).

13 Sheela, T. & Nayaka, Y. A. Kinetics and thermodynamics of cadmium and lead ions adsorption on NiO nanoparticles. *Chemical Engineering Journal* **191**, 123-131 (2012).

14 Ho, Y.-S. Review of second-order models for adsorption systems. *Journal of hazardous materials* **136**, 681-689 (2006).

15 Zeldowitsch, J. Adsorption site energy distribution. *Acta phys. chim. URSS* **1**, 961-973 (1934).

16 Srivastava, V. C., Mall, I. D. & Mishra, I. M. Characterization of mesoporous rice husk ash (RHA) and adsorption kinetics of metal ions from aqueous solution onto RHA. *Journal of hazardous materials* **134**, 257-267 (2006).

17 Chen, S., Yue, Q., Gao, B., Li, Q. & Xu, X. Removal of Cr (VI) from aqueous solution using modified corn stalks: Characteristic, equilibrium, kinetic and thermodynamic study. *Chemical Engineering Journal* **168**, 909-917 (2011).

18 Han, R. *et al.* Characterization of modified wheat straw, kinetic and equilibrium study about copper ion and methylene blue adsorption in batch mode. *Carbohydrate Polymers* **79**, 1140-1149 (2010).

19 Weber, W. J. & Morris, J. C. Kinetics of adsorption on carbon from solution. *Journal of the Sanitary Engineering Division* **89**, 31-60 (1963).

20 Kannan, N. & Sundaram, M. M. Kinetics and mechanism of removal of methylene blue by adsorption on various carbons—a comparative study. *Dyes and pigments* **51**, 25-40 (2001).

21 Chio, C.-P., Lin, M.-C. & Liao, C.-M. Low-cost farmed shrimp shells could remove arsenic from solutions kinetically. *Journal of hazardous materials* **171**, 859-864 (2009).

22 Luo, J. *et al.* Removal of antimonite (Sb (III)) and antimonate (Sb (V)) from aqueous solution using carbon nanofibers that are decorated with zirconium oxide (ZrO2). *Environmental science & technology* **49**, 11115-11124 (2015).

23 Sarı, A., Şahinoğlu, G. n. r. & Tüzen, M. Antimony (III) adsorption from aqueous solution using raw perlite and Mn-modified perlite: equilibrium, thermodynamic, and kinetic studies. *Industrial & engineering chemistry research* **51**, 6877-6886 (2012).

24 Langmuir, I. The constitution and fundamental properties of solids and liquids. Part I. Solids. *Journal of the American chemical society* **38**, 2221-2295 (1916).

25 Freundlich, H. Over the adsorption in solution. *J. Phys. Chem* **57**, 1100-1107 (1906).

26 Alam, M. S. *et al.* Thermodynamic analysis of nickel (II) and zinc (II) adsorption to biochar. *Environmental science & technology* **52**, 6246-6255 (2018).

27 Baes, C. & Mesmer, R. The Hydrolysis of Cations Wiley. *New York*, 177-182 (1976).

28 Filella, M., Belzile, N. & Chen, Y.-W. Antimony in the environment: a review focused on natural waters: II. Relevant solution chemistry. *Earth-Science Reviews* **59**, 265-285 (2002).

29 McComb, K. A., Craw, D. & McQuillan, A. J. ATR-IR spectroscopic study of antimonate adsorption to iron oxide. *Langmuir* **23**, 12125-12130 (2007).

30 Tighe, M., Lockwood, P. & Wilson, S. Adsorption of antimony (V) by floodplain soils, amorphous iron (III) hydroxide and humic acid. *Journal of Environmental Monitoring* **7**, 1177-1185 (2005).
